# Palladium Nanoparticles from *Desulfovibrio alaskensis* G20 Catalyze Biocompatible Sonogashira and Biohydrogenation Cascades

**DOI:** 10.1021/jacsau.2c00366

**Published:** 2022-10-19

**Authors:** Yuta Era, Jonathan A. Dennis, Louise E. Horsfall, Stephen Wallace

**Affiliations:** † Institute of Quantitative Biology, Biochemistry and Biotechnology, School of Biological Sciences, University of Edinburgh, King’s Buildings, Alexander Crum Brown Road, Edinburgh EH9 3FF, U.K.; ‡ EaStCHEM School of Chemistry, University of Edinburgh, King’s Buildings, David Brewster Road, Edinburgh EH9 3FJ, U.K.

**Keywords:** biocompatible chemistry, nanoparticles, cascade, microorganisms, green chemistry

## Abstract

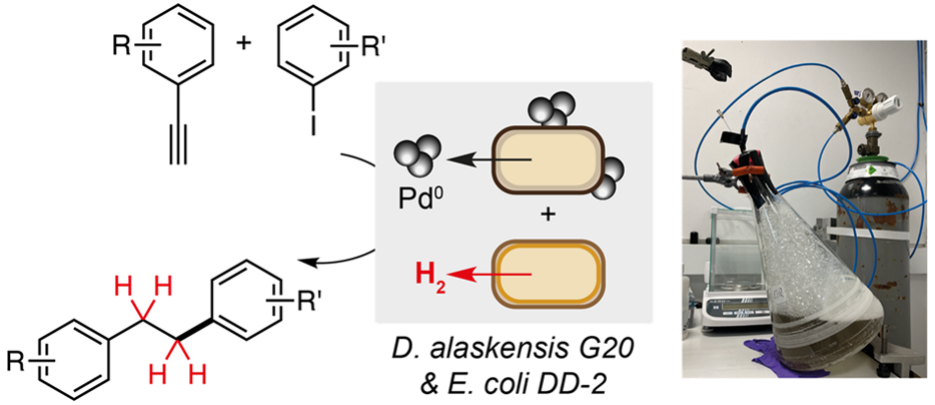

Transition-metal nanoparticles produced by living bacteria are emerging as novel catalysts for sustainable synthesis. However, the scope of their catalytic activity and their ability to be integrated within metabolic pathways for the bioproduction of non-natural small molecules has been underexplored. Herein we report that Pd nanoparticles synthesized by the sulfate-reducing bacterium *Desulfovibrio alaskensis* G20 (*Da*PdNPs) catalyze the Sonogashira coupling of phenyl acetylenes and aryl iodides, and the subsequent one-pot hydrogenation to bibenzyl derivatives using hydrogen gas generated from d-glucose by engineered *Escherichia coli* DD-2. The formal hydroarylation reaction is biocompatible, occurs in aqueous media at ambient temperature, and affords products in 70–99% overall yield. This is the first reported microbial nanoparticle to catalyze the Sonogashira reaction and the first demonstration that these biogenic catalysts can be interfaced with the products of engineered metabolism for small molecule synthesis.

In the absence of oxygen many obligate and facultative anaerobic microorganisms respire using metal ions and small molecules. This includes transition metals such as Pd^2+^ and Pt^2+^, producing zerovalent Pd^0^ and Pt^0^ nanoparticles through dissimilatory metal reduction.^1^ Although not fully characterized, biogenic nanoparticles (NPs) are formed at the inner periplasmic membrane via the reduction of M^*n*+^ to M^0^ by respiratory cytochromes before export and binding to the cell surface.^2^ As the resulting metal nanoparticles are biocompatible, this process enables microbes to thrive in extreme anoxic environments containing toxic metal ions. This is especially efficient in the sulfate-reducing bacterium *Desulfovibrio alaskensis* G20, which generates small, uniformly sized nanoparticles of many platinum group metals in greater than 95% yield under mild conditions.^3^ As such, *D. alaskensis* is under active investigation as a future biotechnology for the remediation of metal waste and leachate from industrial processes and contaminated landfill sites.^4^ However, despite studies into the mechanism of Pd nanoparticle formation and the use of this process for bioremediation, the catalytic chemistry of these bacterial nanoparticles has received little attention. This is despite metal nanoparticles generated by plants and other microorganisms having demonstrated catalytic activity.^1a,5^ To this end, our laboratories recently demonstrated that Pd nanoparticles generated by *D. alaskensis* G20 (*Da*PdNPs) are highly active catalysts for the Suzuki Miyaura cross-coupling of aryl bromides and aryl boronic acids in membrane-bound TPGS micelles (Figure 1A).^6^ These biogenic metal catalysts outperformed other chemically and biologically synthesized Pd nanoparticles from plants and bacteria, highlighting the unique properties of *Da*PdNPs for abiotic catalysis. However, despite the high activity of these microbial Pd catalysts, their use in other C–C bond-forming reactions in vitro and in vivo has yet to be reported. Herein we report that biogenic Pd nanoparticles from *D. alaskensis* G20 catalyze the one-pot, copper-free Sonogashira cross-coupling reaction of phenylacetylenes and aryl iodides, and hydrogenation of the resulting diphenylacetylenes using hydrogen gas produced by engineered *Escherichia coli* DD-2 (Figure 1B). This is the first report of a microbial Pd catalyst for the Sonogashira reaction and the first combined use of these microbially generated, bifunctional Pd catalysts with the products of an engineered metabolic pathway for the synthesis of abiotic small molecules.

**Figure 1:**
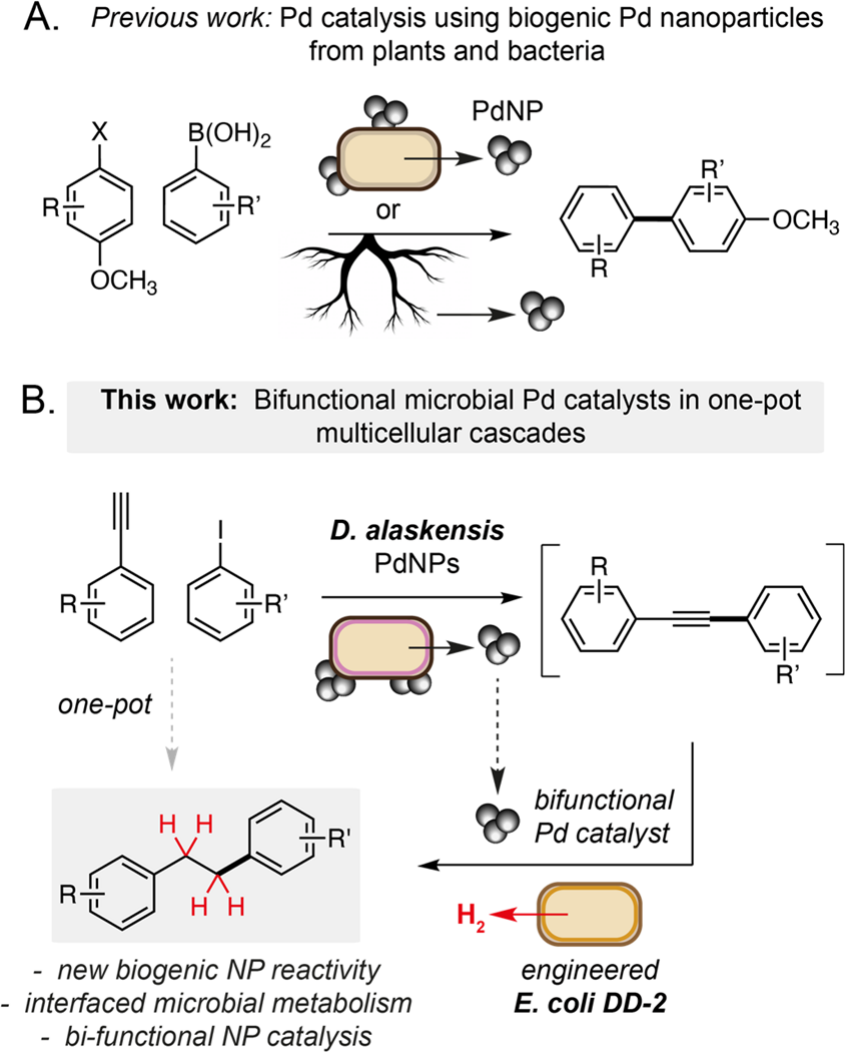
(A) Previous approaches to Suzuki Miyaura cross-coupling using biogenic microbial and plant-derived Pd nanoparticles. (B) Bifunctional Pd nanoparticles from *D. alaskensis* G20 catalyze copper-free Sonogashira reaction and alkyne biohydrogenation reactions.

Our studies began by investigating whether PdNPs from *Desulfovibrio alaskensis* G20 could catalyze the Sonogashira cross-coupling reaction under biorelevant conditions. Palladium nanoparticles were prepared, as reported previously, by anaerobic culturing of *D. alaskensis* G20 in the presence of Na_2_PdCl_4_ followed by centrifugation and Pd quantification by ICP-OES.^6^ Following reports by Lipshutz et al. and ourselves, we chose 4-haloanisoles and phenylacetylene as substrates, tribasic potassium phosphate as the base, and XPhos as the ligand for Pd.^7^ Reactions were conducted in aqueous media in the presence of the Vitamin E-derived surfactant TPGS-1000, which is known to form micelles that co-localize *Da*PdNPs and reactants and associate with the cell membrane. Initial reactions using bromoanisole **1** afforded trace amounts of 1-methoxy-4-(phenylethynyl)benzene **4** by ^1^H NMR. Pleasingly, the use of iodoanisole **2** increased product conversion to 20%. Altering the base to cesium carbonate reduced the yield twofold to 9%; however, the use of triethylamine increased the yield of **4** to 37%. This is in line with previous reports that triethylamine increases the yields of other C–C cross-coupling reactions catalyzed by Pd(P(^*t*^Bu)_3_)_2_, PdCl_2_(dtbpf), and PdCl_2_(CH_3_CN)_2_ in TPGS-750-M and PTS micelles.^7^ Further increasing the concentration of Et_3_N to 90 mM resulted in a moderate increase in **4** to 51% yield. Under these conditions, eliminating the ligand decreased the yield to 27%, so we next screened various phosphine ligands with the aim of further increasing product conversion (Table 1, entries 7–15). The use of the less-substituted RuPhos ligand **8** decreased the yield to 35%, whereas modification of the phosphine substituent on the XPhos ligand from Cy_2_ to (^*t*^Bu)_2_ in ^*t*^BuXPhos **9** had no effect. Significantly altering the electronics of the biphenyl ring through the use of 2,6-dimethoxy groups in SPhos **10** increased the yield to 60%. However, use of the unsubstituted biphenylphosphine ligand JohnPhos **11** increased the yield of **4** to more than 99%, indicating that electronics in addition to ligand planarity was key to increasing the reactivity of *Da*PdNPs in TPGS-1000 micelles. Interestingly, use of the Cy-JohnPhos ligand **12** significantly abolished reactivity, as did the use of the ferrocene-based ligand 1,1′-bis(di-*tert*-butylphosphino)ferrocene (dtbpf) **13**. The simple phosphine ligand PPh_3_**14** decreased the yield to 12%, whereas P(^*t*^Bu)_3_**15** and the Takasago Cy-cBRIDP ligand **16** only moderately decreased the yield of **4** to 77% and 74%, respectively. Although the precise reason(s) for these ligand effects are currently unclear, similar observations have been reported by Jin et al. for the Sonogashira reaction catalyzed by Pd(cinnamyl)(cBRIDP)Cl in TPGS-750-M micelles.^8^ Finally, under these optimized conditions we found that the concentration of Et_3_N could be reduced to 30–45 mM while retaining more than 90% conversion and could also be replaced entirely with K_2_CO_3_ or M9 growth media (Table 1, entries 16–18 and Figure 2B). This latter result was especially promising, as it suggests that the *Da*PdNP-catalyzed Sonogashira reaction could be interfaced with microbial metabolism and developed as a new biocompatible reaction. Together, the combined use of JohnPhos, base, and TPGS-1000 increased product formation 12-fold using microbial Pd nanoparticle catalysts (Table S3). Intriguingly, the use of JohnPhos alone was sufficient to increase the yield of **4** from 9% to 48%, whereas the use of TPGS-1000 in the absence of JohnPhos had no effect (9% yield of **4**; Figure 2C and Table S3). This suggests that JohnPhos directly binds to *Da*PdNPs to form an active Pd complex that is sequestered into the micelle interior. Hydrogen-bonding interactions between the terminal hydroxyl group of TPGS-1000 and cell-surface glycans have been hypothesized to facilitate the Suzuki Miyaura reactivity of *Da*PdNPs.^6^ This new observation indicates that this interaction can be combined with direct activation of Pd at the cell membrane to tune and/or activate the chemistry of biogenic Pd toward new modes of reactivity. Finally, we assessed the scope of the *Da*PdNP-catalyzed Sonogashira reaction under our optimized conditions using a range of alkyne and aryl iodide substrates containing heteroatoms and electron-withdrawing and electron-donating functional groups (Figure 3). Product formation was observed for all substrates in up to 99% yield and improved up to sixfold by the presence of TPGS-1000. This was particularly effective for the coupling of poorly reactive electron-deficient aryl iodides containing *para*-NO_2_ substituents (three- to sixfold increase) and their coupling to heterocyclic 3-ethynylpyridine (Figure 3).

**Figure 2:**
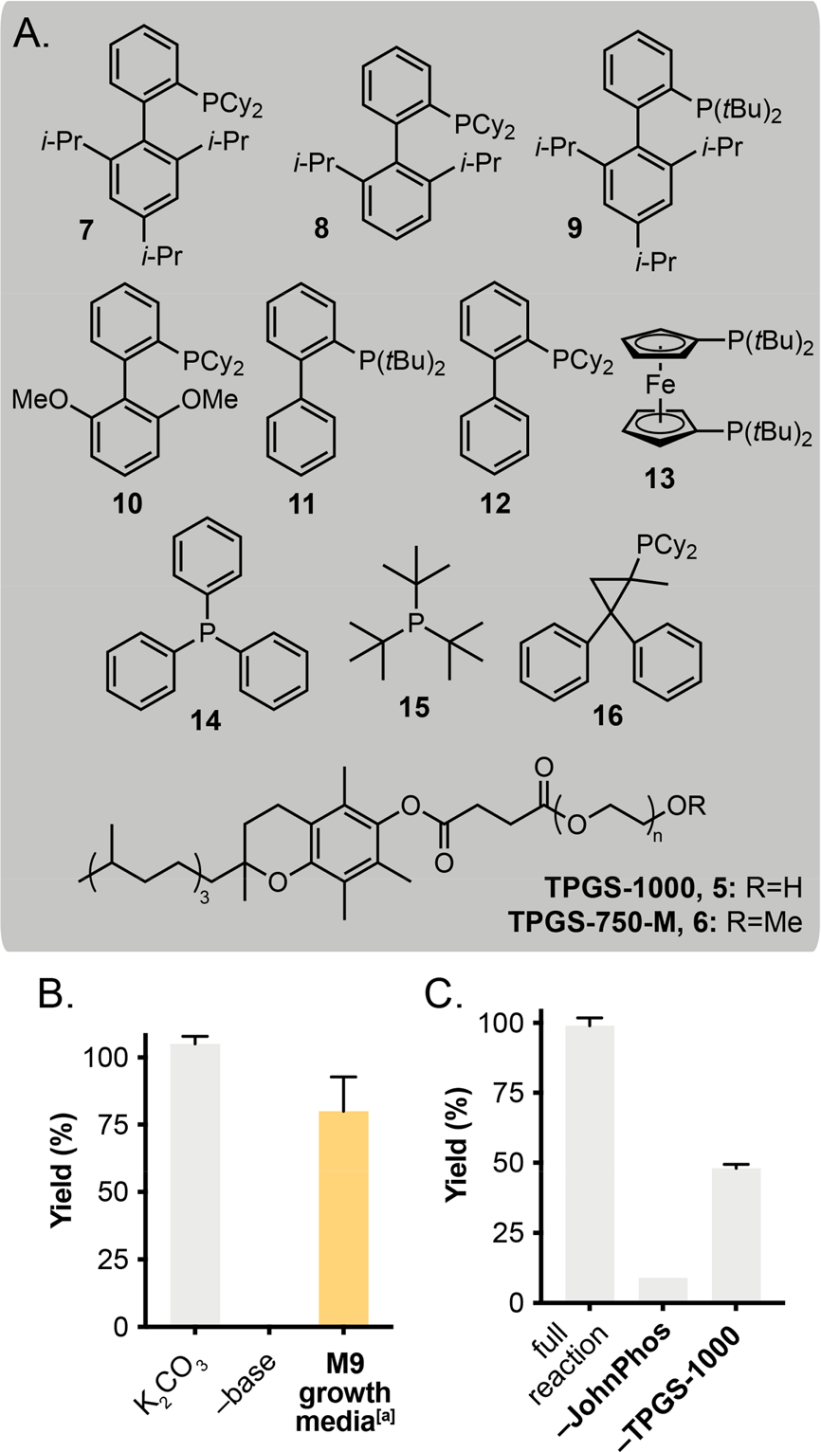
Initial screening data. (A) Structures of phosphine ligands and surfactants. XPhos **7**, RuPhos **8**, ^*t*^BuXPhos **9**, SPhos **10**, JohnPhos **11**, Cy-JohnPhos **12**, dtbpf **13**, PPh_3_**14**, P(^*t*^Bu)_3_**15**, Cy-cBRIDP **16**. For TPGS-750-M, n=17; for TPGS-1000, n=23. (B) Base-free reactivity in M9 growth media. (C) Control reactions examining the components of the reaction. Reactions were set up and analyzed as outlined in Table 1. [a] 5 mM substrate concentration. All data are shown as an average of replicate experiments to one standard deviation.

**Figure 3:**
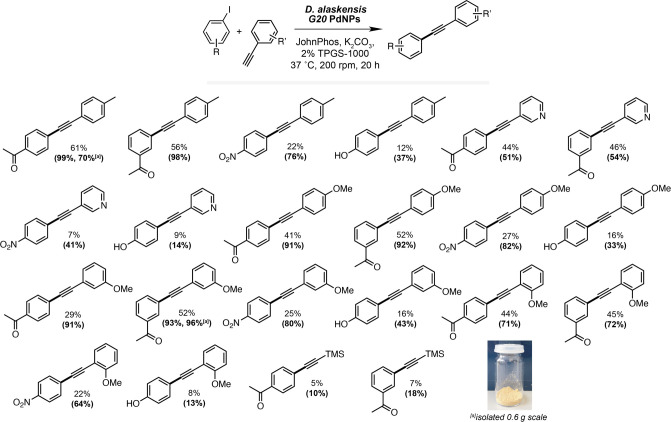
Substrate scope of the *Da*PdNP-catalyzed Sonogashira reaction in the presence and absence of TPGS-1000 micelles. Reactions were performed using aryl iodides (25 mM), alkynes (30 mM), K_2_CO_3_ (30 mM), *Da*PdNPs (0.25 mM, 1 mol %), and JohnPhos ligand (2.5 mM) with/without TPGS-1000 (2% w/vol). Data are reported as percentage yields, and values in parentheses are from reactions containing 2% w/vol TPGS-1000. Product conversions were determined by quantitative ^1^H NMR analysis relative to an internal standard of TMB (10 mM). [a] Isolated yield from 0.6 g scale reaction.

**Table 1:**
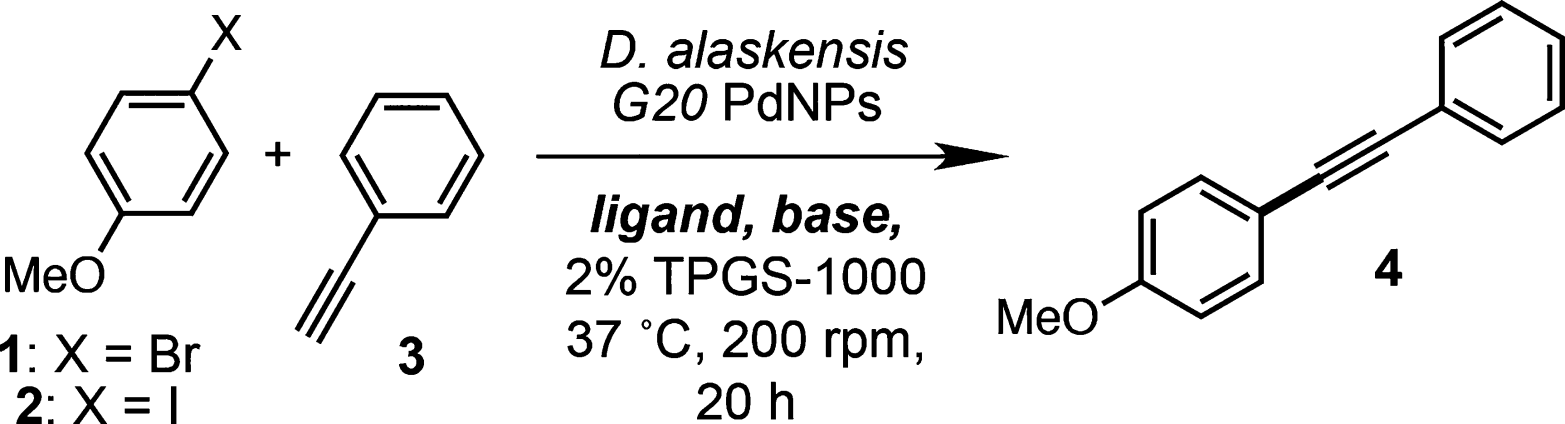
Catalyst, Ligand, and Base Screen for the DaPdNP-Catalyzed Sonogashira Reaction^a^

entry	catalyst	ligand	base	yield (%)
1	*Da*PdNP^b^	**7** (XPhos)	K_3_PO_4_·3H_2_O^d^	<1
2	*Da*PdNP^c^	**7**	K_3_PO_4_·3H_2_O^d^	20
3	*Da*PdNP^c^	**7**	Cs_2_CO_3_^d^	9
4	*Da*PdNP^c^	**7**	Et_3_N^d^	37
5	*Da*PdNP^c^	**7**	Et_3_N^e^	51
6	*Da*PdNP^c^	–	Et_3_N^e^	27
7	*Da*PdNP^c^	**8** (RuPhos)	Et_3_N^e^	35
8	*Da*PdNP^c^	**9** (^*t*^BuXPhos)	Et_3_N^e^	49
9	*Da*PdNP^c^	**10** (SPhos)	Et_3_N^e^	60
10	*Da*PdNP^c^	**11** (JohnPhos)	Et_3_N^e^	>99
11	*Da*PdNP^c^	**12** (Cy-JohnPhos)	Et_3_N^e^	7
12	*Da*PdNP^c^	**13** (dtbpf)	Et_3_N^e^	3
13	*Da*PdNP^c^	**14** (PPh_3_)	Et_3_N^e^	12
14	*Da*PdNP^c^	**15** (P(^*t*^Bu)_3_)	Et_3_N^e^	74
15	*Da*PdNP^c^	**16** (Cy-cBRIDP)	Et_3_N^e^	77
16	*Da*PdNP^c^	**11**	Et_3_N^f^	91
17	*Da*PdNP^c^	**11**	Et_3_N^g^	>99
18	*Da*PdNP^c^	**11**	K_2_CO_3_^f^	94
19	*c*PdNP^c^	**11**	K_2_CO_3_^f^	0
20	Pd/C^c^	**11**	K_2_CO_3_^f^	43

a Reactions were performed using **1** or **2** (30 mM), **3** (60 mM), base, Pd catalyst (0.3 mM, 1 mol %), ligand (3 mM), and TPGS-1000 (2% w/vol) in sealed tubes under an atmosphere of air.

b Aryl bromide **1** was used as the substrate

c Aryl iodide **2** was used as the substrate.

d 60 mM.

e 90 mM.

f 30 mM.

g 45 mM. *c*PdNPs refers to nanoparticulate Pd black (<25 nm particle size). Product concentrations were determined by ^1^H NMR spectroscopy relative to an internal standard of TMB. All data are shown as an average of replicate experiments to one standard deviation.

We next proceeded to assess whether the *Da*PdNP-catalyzed Sonogashira reaction could proceed in the presence of a living microorganism and be interfaced with engineered metabolism. The field of biocompatible chemistry is an emerging approach in chemical biotechnology that aims to expand the biosynthetic scope of living organisms by interfacing nonenzymatic chemical catalysis with native and engineered metabolic pathways. In doing so, chemical tools can not only be employed to direct metabolic function but also diversify metabolic output, enabling synthetic biology approaches to be used to produce non-natural compounds of industrial importance that cannot be accessed by enzymes alone. Recent work in this field includes the use of InP nanoparticles to enable cofactor recycling in *Saccharomyces cerevisiae*,^9^ Fe carbene-transfer catalysis to enable cyclopropane formation from d-glucose in *E. coli*,^10^ and amine organocatalysis to enable the aldol dimerization of metabolic aldehydes in *Gluconobacter oxidans*.^11^ Inspired by this and our own work in this area, we envisioned that the *Da*PdNP-catalyzed Sonogashira reaction could be a good candidate biocompatible reaction; product formation is facile under aqueous conditions and can occur in microbial growth media, and diphenylacetylenes cannot be synthesized using known enzymes. Motivated by seminal work by Balskus et al., we chose to examine whether the Sonogashira reaction could be interfaced with metabolic H_2_(g) in a biocompatible alkyne hydrogenation reaction.^12^ This would enable access to bibenzyl products in a one-pot formal hydroarylation reaction while also assessing the ability of *Da*PdNPs to perform two catalytic reactions simultaneously. We chose to use the strain *E. coli* DD-2, an engineered H_2_ overproducer first reported by Silver et al. containing plasmids encoding for the inducible expression of a pyruvate ferredoxin oxidoreductase, a ferredoxin, and an [Fe–Fe] hydrogenase.^13^ Biocompatible alkene hydrogenation has been reported using this strain and the Royer Pd catalyst (Pd on polyethylenimine-SiO_2_) but not using biological sources of Pd or in tandem catalytic reactions. To this end, we confirmed the biocompatibility of the reaction components to *E. coli* DD-2 by incubating cells in the presence of *Da*PdNPs, diphenylacetylene, and TPGS-1000 at mid log phase growth (OD_600_ = 0.5–0.6) and observing only a 10-fold decrease in the number of viable cells after 18 h by serial dilution and plate-count assays (Figures S8 and S9). Replicating this experiment under anaerobic growth conditions using *E. coli* DD-2 and diphenylacetylene as a substrate resulted in 60% conversion to bibenzyl, indicating that *Da*PdNPs were active hydrogenation catalysts and that the product of the Sonogashira reaction was a viable substrate for biohydrogenation. Having confirmed that the *Da*PdNP-catalyzed Sonogashira reaction occurs in M9 growth media and that the product can be hydrogenated using *Da*PdNPs and microbial hydrogen gas, we next moved on to combine these reactions into a one-pot process. A 9:1 mixture of M9/M9CA growth media (M9-glucose +10% CA) was found to be necessary for both reactions to occur in greater than 80% yield, as hydrogen gas formation in *E. coli* DD-2 requires cell growth in M9CA media and *Da*PdNPs are inhibited by high concentrations of casamino acids (Tables S6 and S7). Pleasingly, inoculation of an overnight culture of *E. coli* DD-2 to the Sonogashira reaction after 44 h resulted in cell growth, hydrogen production, and hydrogenation of diphenylacetylene to bibenzyls **19** in 71–76% overall yield (Figure 4). The product could also be isolated in 65% yield from a 0.3 g scale reaction (Figures 4A and S10). *cis-* and *trans-*Stilbene isomers **18** were detected in reactions in ca. 30% combined yield, leading to the hypothesis that catalyst deactivation was occurring after prolonged reaction times. Indeed, spiking reactions after 5 d with freshly prepared *Da*PdNPs consumed the residual stilbene and increased the yield of bibenzyl to 91% (Figure S6). Overall, we hypothesize that the initial Sonogashira reaction occurs in membrane-associated TPGS micelles containing an active *Da*PdNP-JohnPhos complex, followed by anaerobic growth of *E. coli* DD-2 in the surrounding medium, hydrogen gas formation, and *Da*PdNP-catalyzed alkyne reduction in micelles.

**Figure 4:**
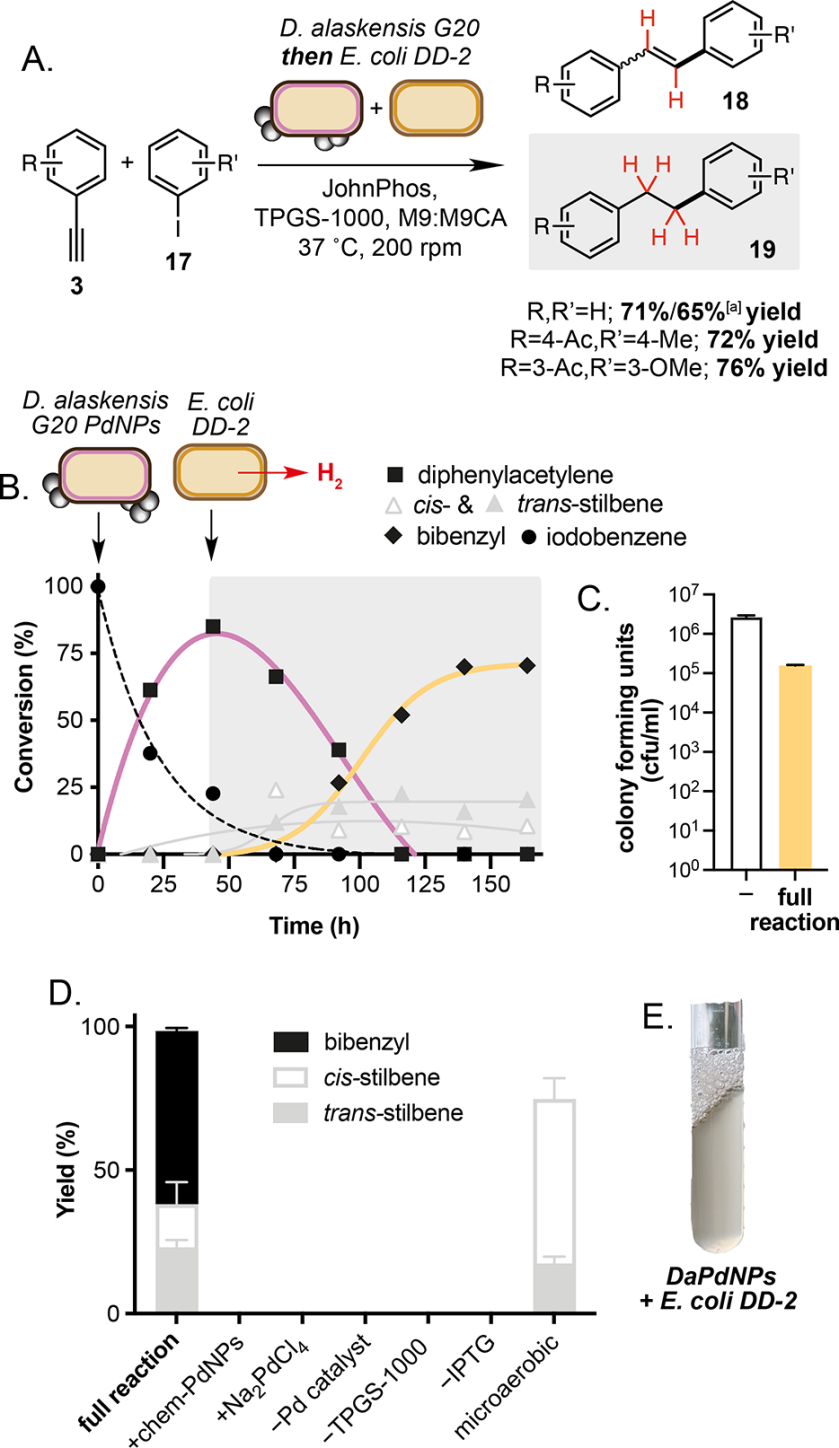
One-pot Sonogashira and biohydrogenation cascade using *Da*PdNPs and *E. coli* DD-2. (A) The one-pot reaction involving *Da*PdNP-catalyzed cross-coupling followed by addition of *E. coli* DD-2 and biohydrogenation. (B) Product formation during the reaction. Addition of *Da*PdNPs and *E. coli* DD-2 (1/100 inoculum of a saturated overnight culture grown in M9-glucose +10% CA containing antibiotics) are indicated by black arrows. Error bars are omitted for clarity. (C) Plate count assay to determine cell viability during the reaction. (D) Control experiments showing that product formation requires microbial Pd catalyst and *E. coli* DD-2 in addition to micelles, ligand, and anaerobic growth conditions. (E) Photograph of the biohydrogenation reaction. Reactions were performed in *E. coli* DD-2 (OD_600_ = 0.5) culture in M9-glucose +10% CA media using diphenylacetylene (1 mM), *Da*PdNPs (0.25 mM), IPTG (0.5 mM), Fe(NH_4_)_2_(SO_4_)_2_ (50 μM), antibiotics, and TPGS-1000 (2% w/vol) in sealed tubes under an anaerobic atmosphere. All data are shown as an average of three independent experiments to one standard deviation. Product concentrations were determined by ^1^H NMR relative to an internal standard of TMB (2–5 mM). [a] Isolated yield from 0.3 g scale reaction.

A series of control reactions confirmed that product formation was dependent on the presence of *Da*PdNPs, ligand, and micelles (Figure 4D). No product conversion was observed in the presence of chemically synthesized Pd nanoparticles or NaPdCl_4_, confirming the unique reactivity of microbial *Da*PdNPs in this tandem catalytic reaction.^14^ Microbial H_2_ was confirmed as the reductant for the hydrogenation by eliminating isopropyl-β-d-thiogalactoside (IPTG) and thus H_2_ biosynthesis and observing no product formation. This experiment also eliminated the possibility that product formation occurs by transfer hydrogenation of diphenylacetylene and/or stilbene isomers by a Pd hydride formed from formate in *E. coli*. Conducting the reaction under microaerobic conditions inhibited hydrogen gas formation in part and afforded *cis-* and *trans-*stilbenes as sole products in 75% yield and 3.3:1 ratio, respectively. Limiting hydrogen gas formation in vivo by altering the culture headspace concentration of O_2_ can therefore be used to achieve alkene products akin to a Lindlar reduction using Pd/BaSO_4_, enabling further downstream functionalization (Figure 4D).

Finally, imaging the cells by transmission electron microscopy confirmed the presence of intact cells bound within a micellar matrix (Figures 5 and S11). Distinct interactions were observed between *E. coli* and *D. alaskensis* Pd nanoparticles, and these interactions were absent in images taken of sampled cultures grown in the absence of TPGS-1000 (Figures S12 and S13). This supports the hypothesis that both the Pd-catalyzed Sonogashira and hydrogenation reactions likely occur in membrane-associated micelles and that cell contact is not necessary but enhanced in the presence of TPGS-1000.

**Figure 5:**
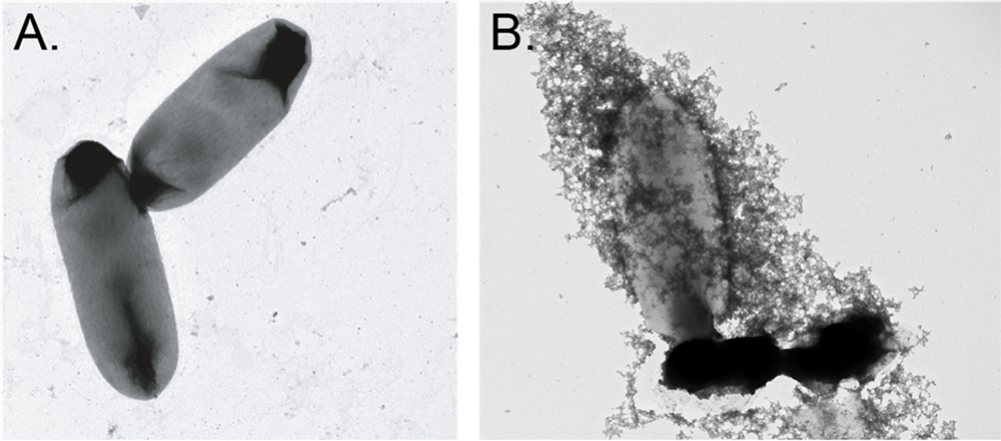
Imaging the reaction by transmission electron microscopy. (A) *E. coli* DD-2. (B) *E. coli* DD-2, *Da*PdNPs, and TPGS-1000.

In summary, we have reported that biogenic Pd nanoparticles generated by the anaerobic bacterium *Desulfovibrio alaskensis* G20 catalyze the Sonogashira cross-coupling of phenylacetylenes and aryl iodides in membrane-associated TPGS micelles. The reaction occurs under mild conditions (aqueous media, 37 °C, pH 7.4) using a range of substrates, outperforming other heterogeneous Pd catalysts generated via chemical methods and aligning well to reported green chemistry methods. These biogenic Pd nanoparticles can also be interfaced with a hydrogen-producing strain of *Escherichia coli* to enable the one-pot synthesis of bibenzyl derivatives. To the best of our knowledge, this is the first example of a microbial Pd catalyst able to catalyze the Sonogashira reaction and the first use of a bifunctional biogenic metal nanoparticle as a biocompatible catalyst with engineered *E. coli*. It is our opinion that the combined use of transition metal catalysts from bacteria with new ligands and surfactants from the field of organic chemistry will continue to enable the sustainable synthesis of novel compounds that are currently inaccessible to engineered biological systems.

## Methods

### Bioproduction of Pd Nanoparticles

*Desulfovibrio alaskensis* G20 (DSM 17464) was grown to OD_600_ 0.3 in Postgate media C (50 mL), recovered via centrifugation, and washed with MOPS buffer before resuspension to OD_600_ 1.0 in 50 mL centrifuge tubes containing 40 mL of MOPS buffer. Na_2_PdCl_4_ (80 μmol) was added, and cells were incubated statically at 30 °C for 20 h in an anaerobic chamber. The biogenic nanoparticles (*Da*PdNPs) were harvested by centrifugation (15 min, 4500*g*) and washed with 1:1 acetone/H_2_O (40 mL). The resulting *Da*PdNPs were freeze-dried, resuspended in deionized water, and sonicated in a water bath for 30 min prior to analysis by TEM and quantification of Pd by ICP-OES.

### Small-Scale Sonogashira Reaction

To a dried 15 mL Hungate tube containing 5 mL of deionized water, *Da*PdNPs (1.5 μmol), haloanisole (0.15 mmol), phenylacetylene (0.3 mmol), ligand (15 μmol), base (0.3–0.9 mmol) and surfactant (0.1 g) were added. The tube was sealed with a rubber septum and a screw-cap and incubated at 37 °C (200 rpm) for 20 h. After this time, the reaction was extracted with dichloromethane and concentrated under reduced pressure. The crude residue was dissolved in 1 mL of CDCl_3_ containing 1,3,5-trimethoxybenzene (TMB) (10 μmol), dried over anhydrous Na_2_SO_4_ and analyzed by ^1^H NMR spectroscopy.

### Preparative-Scale Sonogashira Reaction

To a dried 250 mL flask containing 100 mL of deionized water, *Da*PdNPs (25 μmol), aryl iodide (2.5 mmol), alkyne (3 mmol), JohnPhos (0.25 mmol), K_2_CO_3_ (3 mmol) and TPGS-1000 (2.0 g) were added. The flask was sealed with a silicone rubber septum and incubated at 37 °C (200 rpm) for 20 h. After this time, the reaction was extracted with dichloromethane, filtered to remove *Da*PdNPs, dried over anhydrous Na_2_SO_4_ and concentrated under reduced pressure. The crude product was purified by column chromatography on silica gel using hexanes and ethyl acetate.

### Biohydrogenation

To a dried 15 mL Hungate tube containing 5 mL of *E. coli* DD-2 in M9CA media (grown to OD_600_ 0.5–0.6), *Da*PdNPs (1.25 μmol), TPGS-1000 (0.1 g), IPTG (2.5 mmol), Fe(NH_4_)_2_(SO_4_)_2_ (0.25 mmol) and appropriate antibiotics were added. The tube was sealed with a rubber septum and a screw-cap, and the reaction was sparged with nitrogen gas for 10 min. A solution of diphenylacetylene (5 μmol in EtOH) was added and the culture was incubated at 37 °C (200 rpm) for the appropriate time. The reaction was diluted with 5 mL of brine and a 1 mL aliquot extracted into 1 mL of CDCl_3_ containing TMB (10 μmol). The organic extract was dried over anhydrous Na_2_SO_4_ and analyzed by ^1^H NMR spectroscopy.

### Small-Scale Sonogashira/Biohydrogenation Cascade

To a dried 15 mL Hungate tube containing 5 mL of M9-glucose (+0.2 g/L casamino acids), *Da*PdNPs (1.25 μmol), JohnPhos (12.5 μmol) and TPGS-1000 (0.1 g) were added. The tube was sealed with a rubber septum and a screw-cap, and the reaction was sparged with nitrogen gas for 10 min. A 1:1 solution of iodobenzene and phenylacetylene (5 μmol in EtOH) was added and the reaction was incubated at 37 °C (200 rpm) for 44 h. After this time, 0.5 mL of *E. coli* DD-2 (grown to OD_600_ 0.5–0.6 in 6 mL of M9CA and concentrated to OD_600_ 6.0–7.2) and fresh M9CA (0.5 mL, containing IPTG (3.0 mmol), Fe(NH_4_)_2_(SO_4_)_2_ (0.3 mmol) and antibiotics) were added and the culture was incubated for a further 120 h. After this time, the culture was diluted with 6 mL of brine and a 1 mL aliquot extracted into 1 mL of CDCl_3_ containing TMB (2–5 μmol). The organic extract was dried over anhydrous Na_2_SO_4_ and analyzed by ^1^H NMR spectroscopy.

### Preparative-Scale Sonogashira/Biohydrogenation Cascade

To a dried 4 L Erlenmeyer flask containing 1.5 L of M9-glucose (+ 0.2 g/L casamino acids), *Da*PdNPs (0.38 mmol), JohnPhos (3.75 mmol), and TPGS-1000 (30 g) were added. The flask was sealed with a silicone rubber septum and the reaction was sparged with nitrogen gas for 30 min. Iodobenzene (1.5 mmol) and phenylacetylene (1.5 mmol) were added and the flask was incubated at 37 °C (200 rpm) for 44 h. After this time, 50 mL of *E. coli* DD-2 (grown to OD_600_ 0.5–0.6 in 1.6 L of M9CA and concentrated to OD_600_ 16–19) and fresh M9CA (50 mL, containing IPTG (0.8 mol), Fe(NH_4_)_2_(SO_4_)_2_ (80 mmol) and antibiotics) were added and the culture was incubated for a further 120 h. After this time, NaCl (263 g) was added and the reaction was extracted with chloroform. The combined organic extracts were filtered to remove *Da*PdNPs, dried over anhydrous Na_2_SO_4_ and concentrated under reduced pressure. The crude product was purified by column chromatography on silica gel using hexanes.

## Supporting Information Available

Synthetic procedures for nanoparticle bioproduction and cross-coupling/biohydrogenation cascades are available, including ^1^H/^13^C NMR and HRMS characterization data (PDF)
