# Supplementary material for: Palladium Nanoparticles from Desulfovibrio alaskensis G20 Catalyze Biocompatible Sonogashira and Biohydrogenation Cascades
Source: JACS Au. 2022 Oct 19;2(11):2446–52. doi: 10.1021/jacsau.2c00366 (PMC9709939; doi:10.1021/jacsau.2c00366)
Supplement: Supplementary file 1 — au2c00366_si_001.pdf [file au2c00366_si_001.pdf]

SUPPLEMENTARY INFORMATION

Palladium nanoparticles from *Desulfovibrio alaskensis* G20 catalyze biocompatible Sonogashira and bio-hydrogenation cascades

Yuta Era[1], Jonathan A. Dennis[1,2], Louise E. Horsfall[1]* and Stephen Wallace[1]*

[1]Institute of Quantitative Biology, Biochemistry and Biotechnology, School of Biological Sciences, University of Edinburgh, Roger Land Building, Alexander Crum Brown Road, King's Buildings, Edinburgh, EH9 3FF

[2]School of Chemistry, University of Edinburgh, Joseph Black Building, David Brewster Road, King's Buildings, Edinburgh, EH9 3FF

*Correspondence to: stephen.wallace@ed.ac.uk; louise.horsfall@ed.ac.uk

Supplementary Materials

S1 General materials and methods S3

S2 Strain, media and culture condition... S4

S3 *Da*PdNPs production by *Desulfovibrio alaskensis* S5

S4 General Sonogashira coupling reaction procedure for initial optimization........................ S5

S5 Coupling reaction optimization in aqueous solution S6

Table S1 *Reaction screen*

S6 Coupling reaction with other Pd catalysts.. S7

Table S2 *Coupling reaction in the presence of other Pd catalysts*

S7 Negative control experiments.. S8

Table S3 *Effect of JohnPhos and TPGS-1000 on reaction yield*

Figure S1. *Effect of JohnPhos and TPGS-1000 on reaction yield*

S8 Reaction scope investigation of Sonogashira coupling S9

Table S4 *Reaction scope of Sonogashira reation*

S9 Base-free Sonogashira coupling reaction.. S11

Table S5 *Base-free Sonogashira coupling reaction*

S10 Media screen for biocompatible Sonogashira coupling reaction....................................... S11

Table S6 *Biocompatible Sonogashira coupling reaction*

Figure S2. *Control experiments – biocompatible Sonogashira reaction*

S11 Media screen for bio-hydrogenation of diphenylacetylene... S13

Table S7 *Bio-hydrogenation reaction*

Figure S3. *Control experiments – bio-hydrogenation reaction*

S12 Sonogashira/hydrogenation cascade reaction in two tube system..................................... S14

Figure S4. *Scheme of Sonogashira/hydrogenation cascade reaction in two tube system*

Table S8 *Sonogashira/hydrogenation cascade reaction in two tube system*

S13 One-pot Sonogashira/hydrogenation tandem reaction... S15

Figure S5. *Scheme of one-pot Sonogashira/hydrogenation tandem reaction*

Table S9 *One-pot Sonogashira/hydrogenation tandem reaction*

Table S10 *One-pot Sonogashira/hydrogenation tandem reaction (followed by extra DaPdNPs addition)*

Figure S6. *Reaction time-course.*

Figure S7. *^{1}H NMR spectra of crude extract of one-pot Sonogashira/hydrogenation tandem reaction at different time points*

S14 Reaction scope investigation of one-pot Sonogashira/hydrogenation cascade S18

Table S11 *Reaction scope investigation of Sonogashira/hydrogenation tandem reaction*

S15 Cell viability in Sonogashira/hydrogenation cascade S18

Figure S8. *Plate count assay*

Figure S9. *Growth curve assay*

S16 Preparative scale reaction S20

Figure S10. *Preparative scale one-pot Sonogashira/hydrogenation cascade*

Table S12 *Composition of isolated product in preparative scale one-pot Sonogashira/hydrogenation cascade*

Table S13 *Pd content in isolated products from preparative scale reactions*

S17 TEM analysis of *Da*PdNPs, *E. coli* DD-2 and TPGS-1000.. S22

Figure S11. *Negative stained DaPdNPs and E. coli DD-2 in the presence of 0.4% w/vol TPGS-1000*

Figure S12. *Negative stained DaPdNPs and E. coli DD-2 in the absence of TPGS-1000*

Figure S13. *Negative stained E. coli DD-2*

S18 Product characterization.. S24

S19 References S34

S1 General materials and methods

Unless otherwise noted, starting materials and reagents were obtained from commercial suppliers and were used without further purification. All water used experimentally was purified with a Suez Select purification system (18 mΩ/cm, 0.2 μM filter).

NMR: Proton nuclear magnetic resonance spectra (^{1}H NMR) were recorded using an AVA/PRO 500 NMR spectrometer (Bruker) at 500 MHz at 298K. Proton chemical shifts are expressed in parts per million (ppm, δ scale) and are referenced to residual protium in the NMR solvent ($CDCl_3$, δ 7.26 ppm). Coupling constants, *J*, are measured to the nearest 0.1 Hz and are presented as observed. Data is represented as: chemical shift, integration, multiplicity (s = singlet, d = doublet, t = triplet, q = quartet, dd = doublet of doublets, m = multiplet and/or multiple resonances), coupling constant (J) in Hertz. NMR solvents were used as purchased from commercial suppliers. For all quantitative NMR measurements, 1,3,5-trimethoxybenzene (TMB) was used as an internal standard. Carbon nuclear magnetic resonance spectra (^{13}C NMR) were recorded using an AVA500 spectrometer at the specified frequency at 298 K. Chemical shifts are quoted in parts per million (ppm, δ scale) and are referenced to the carbon resonances of the NMR solvent ($CDCl_3$, δ 77.2 ppm).

Chromatography: Analytical thin layer chromatography (TLC) was performed using aluminum plates pre-coated with silica gel impregnated with a fluorescent indicator F_{254} (60 Å pore-size, 230–400 mesh, Supelco). TLC plates were visualized by exposure to ultraviolet light. Column chromatography was performed using Merck Kieselgel 60.

ICP-OES: Inductively Coupled Plasma Optical Emission Spectrometry (ICP-OES) was performed using an Optima 8300 instrument (Perkin Elmer). Experiments were performed using the following conditions: samples were sonicated for 30 min at 21 °C in a water bath and then centrifuged (20,000 × g, 2 h). The supernatant (the ionic fraction) was added to a solution of aqua regia ($HCl:HNO_3$ = 14:10%) and heated to 80 °C for 8 h. The resulting sample was diluted in 2% nitric acid to a final volume of 3 mL and used immediately. Unspun samples (whole fraction) were prepared using an analogous method.

TEM: Transmission electron microscopy (TEM) was performed using a JEM-1400 Plus (JEOL) with an accelerating voltage of 80 kV. TEM images were captured using a GATAN OneView camera. Image processing was carried out using ImageJ software (National Institutes of Health, USA). Experiments were performed using the following conditions: suspensions of *Da*PdNPs were drop cast on to a 200-mesh carbon-coated copper grid and dried for 5 min under air. The excess liquid was then removed and the samples visualized immediately. using a TEM (JEM-1400 Plus, JEOL) with an accelerating voltage of 80 kV.

S2 Strain, media, buffer and culture condition

Desulfovibrio alaskensis G20 (DSM 17464) was obtained from DSMZ. *Desulfovibrio alaskensis* G20 (DSM 17464) was grown statically on de-gassed Postgate Medium C at 30 °C in an anaerobic chamber fed with 10% H_2 and 10% CO_2 in nitrogen.

Engineered hydrogen-producing strain *Escherichia coli* DD-2 was constructed from the parental *E. coli* BL21(DE3) Δ*tonA* strain by Silver et al. previously.[1] In brief, the parental strain was transformed with a modified pCDF-duet containing codon-optimized hydrogenase maturation factors HydEF (multiple cloning site 1) and HydG (multiple cloning site 2) from *Chlamydomonas reinhardtii*, a modified pACYC-duet vector containing pyruvate ferredoxin oxidoreductase from *Desulfovibrio africanus* (multiple cloning site 2), and a modified pET-duet vector encoding artificial fusion protein consisting of [Fe-Fe] hydrogenase and ferredoxin from *Clostridium acetylbutylicum* connected at the hydrogenase C-terminus via a $(Gly_4Ser)_2$ amino acid linker. DD-2 strain was obtained from Prof. Emily Balskus (Harvard University, Boston, MA) and for routine cultivation was grown aerobically at 37 °C on Lysogeny Broth (LB) agar in the presence of ampicillin (50 μg/mL), spectinomycin (25 μg/mL), and chloramphenicol (12.5 μg/mL).

Postgate Medium C was prepared according to the following procedure: KH_2PO_4 (0.5 g), NH_4Cl (1.0 g), Na_2SO_4 (4.5 g), $CaCl_2 \cdot 6H_2O$ (0.06 g), $MgSO_4 \cdot 7H_2O$ (0.06 g), 60% sodium lactate (10 mL) yeast extract (1.0 g), $FeSO_4 \cdot 7H_2O$ (0.004 g) and sodium citrate$\cdot 2H_2O$ (0.3 g) were dissolved in 990 mL ultrapure water and its pH was adjusted to 7.5 using 2 M NaOH. The medium was autoclaved at 121 °C for 20 min and cooled to room temperature. Autoclaved Postgate Medium C was stored at room temperature and degassed in the anaerobic chamber overnight before the use.

M9-glucose media was prepared according to the following procedure: Na_2HPO_4 (3.0 g), KH_2PO_4 (1.5 g), NH_4Cl (0.5 g) and NaCl (0.25 g) were dissolved in 450 mL of ultrapure water and autoclaved at 121 °C for 20 min. Upon cooling to room temperature, 1 mL of 1.0 M aqueous $MgSO_4$, 50 μL of 1.0 M aqueous $CaCl_2$, and 12.5 mL of aqueous 20% (w/vol) glucose were added and the total volume was adjusted to 500 mL using sterile ultrapure water. M9CA-glucose media was prepared similarly: Na_2HPO_4 (3.0 g), KH_2PO_4 (1.5 g), NH_4Cl (0.5 g), NaCl (0.25 g) and casamino acids (2.5 g) were dissolved in 450 mL of ultrapure water and autoclaved at 121 °C for 20 min. Upon cooling to room temperature, 50 μL of aqueous 10 mg/mL thiamine hydrochloride, 1 mL of 1.0 M aqueous $MgSO_4$, 50 μL of 1.0 M aqueous $CaCl_2$, 12.5 mL of aqueous 20% (w/vol) solution of glucose were added, and the total volume was adjusted to 500 mL using sterile water. M9—glucose +10% CA was made by mixing M9-glucose and M9CA-glucose at 9:1 before its use. Both the M9-glucose media and the M9CA-glucose media were stored at room temperature.

MOPS (3-(*N*-morpholino)propanesulfonic acid) buffer was prepared according to the following procedure: MOPS free acid (41.86 g) was dissolved in 1 L ultrapure water and its pH was adjusted to 7.0 using 1 M H_2SO_4. The buffer was autoclaved at 121 °C for 20 min and cooled to room temperature. Autoclaved MOPS buffer was stored at room temperature and degassed in the anaerobic chamber overnight before the use.

Antibiotics, isopropyl β-D-1-thiogalactopyranoside (IPTG) and $Fe(NH_4)_2(SO_4)_2$ were prepared as stock solutions (500−1000 times concentrated) and filter sterilized before use. Optical densities of cultures were determined using a WPA CO8000 spectrophotometer (Biochrom) by measuring absorbance at 600 nm.

S3 *Da*PdNPs production by *Desulfovibrio alaskensis*

D. alaskensis G20 (DSM 17464) was pre-grown following previously reported methods,[2] recovered and washed with MOPS buffer three times prior to inoculation (to a final OD_{600} of 1.0) in 50 mL centrifuge tubes containing 40 mL MOPS buffer. Freshly made Na_2PdCl_4 stock solution (40 mM in H_2O) was added to the cell suspension to a final concentration of 2 mM. The centrifuge tubes were incubated at 30 °C for 20 h in an anaerobic chamber. The biogenic nanoparticles (*Da*PdNPs) were harvested by centrifugation (15 min, 4,500 × g) and washed with 50% acetone in H_2O (1 x vol equiv.). Subsequently, the *Da*PdNPs were freeze-dried overnight, resuspended in ultrapure water and sonicated in a water bath for 30 min. The resulting nanoparticles were analyzed by TEM. The recovery of Pd as *Da*PdNPs was determined by ICP-OES as outlined in S1.

S4 General Sonogashira coupling reaction procedure for initial optimization

Cross coupling reactions were carried out using the following procedure: *Da*PdNPs were added to a 15 mL Hungate tube containing aryl halide (25/30 mM), phenylacetylene (30/60 mM), base and surfactant (2% w/vol) in 5 mL H_2O. The tubes were sealed with butyl rubber septa and screw-caps and incubated at 37 °C (200 rpm) for 20 h in a New Brunswick Innova 44 incubator shaker (Eppendorf). After this time, the reactions were cooled to room temperature, extracted with dichloromethane (DCM, 3 x 1.7 mL) and concentrated under reduced pressure. The crude residue was dissolved in 1 mL $CDCl_3$ containing 10 mM TMB and analyzed by ^1H NMR spectroscopy.

S5 Coupling reaction optimization in aqueous solution

Unless otherwise noted, model reaction (*Da*PdNPs (0.3 mM), halide (30 mM), phenylacetylene (60 mM), base and surfactant (2% w/vol) in H_2O (5 mL)) was used by following the above protocol for reaction optimization and screening experiments.

For halide screening, 4-bromoanisole or 4-iodoanisole were used as the aryl halide. For base screening, $K_3PO_4{\cdot}3H_2O$, Cs_2CO_3, Et_3N, ${}^{i}Pr_2NEt$ or K_2CO_3 was added to the model reaction at various concentration (1.0, 1.5, 2.0 or 3.0 eq to halide concentration). For ligand screening, the model reaction was performed in the presence of XPhos, RuPhos, ^{t}BuXPhos, SPhos, JohnPhos, Cy-JohnPhos, 1,1′-Bis(di-tert-butylphosphino)ferrocene (dtbpf), triphenylphosphine (PPh_3), tri-*tert*-butylphosphine ($P({}^{t}Bu)_3$) or Cy-cBRIDP or in the absence of ligands. For surfactant screening, TPGS-1000, TPGS-750-M, PS-750-M or PTS was added to the model reaction at 2% w/vol. The reactions were conducted and analyzed by following the protocol outlined in Section S4.

Table S1 *Reaction screen*

#	Ar-X	Ar-C≡CH	Ligand	Base (eq.)	Product	
					%conversion	±SD
1	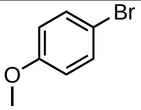 (30 mM)	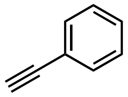 (60 mM)	XPhos	$K_3PO_4{\cdot}3H_2O$ (2.0)	N.D.	N.A.
2	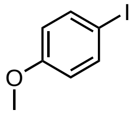 (30 mM)				20	1.7
3				Cs_2CO_3 (2.0)	9	1.3
4				Et_3N (2.0)	37	1.7
5			–	Et_3N (3.0)	27	3.6
6			XPhos		51	0.9
7			RuPhos		35	0.14
8			^{t}BuXPhos		49	1.3
9			SPhos		60	1.3
10			JohnPhos		>99	5.5
11			Cy-JohnPhos		7	1.9
12			dtbpf		3	0.51
13			PPh_3		12	3.9
14			$P({}^{t}Bu)_3$		74	36.2
15			Cy-cBRIDP		77	12.6
16			JohnPhos	Et_3N (1.0)	91	1.8
17				Et_3N (1.5)	>99	0.30

18				Et_3N (2.0)	>99	1.6
19				$^{i}Pr_2NEt$ (1.0)	83	3.8
20				$^{i}Pr_2NEt$ (2.0)	>99	3.8
21				$K_3PO_4{\cdot}3H_2O$ (1.0)	93	2.9
22				$K_3PO_4{\cdot}3H_2O$ (2.0)	>99	2.3
23				K_2CO_3 (1.0)	94	0.49
24				K_2CO_3 (2.0)	>99	0.26
25[a]	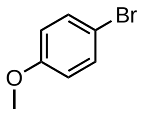 (25 mM)	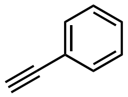 (30 mM)	JohnPhos	K_2CO_3 (1.25)	20	2.5
26[b]					16	14.0
27[c]					3	0.26
28[d]					N.D.	N.A.

N.D.: Not detected, N.A.: Not applicable. Product concentrations were determined by ^{1}H NMR relative to an internal standard of TMB (10 mM). [a] 2% w/vol TPGS-1000, [b] 2% w/vol TPGS-750-M, [c] 2% w/vol PS-750-M, [d] 2% w/vol PTS

S6 Coupling reaction with other Pd catalysts

Chemically produced Pd nanoparticle (<25 nm, ≥99.5%, Sigma-Aldrich product no. 686468) (*c*PdNP), Pd on activated carbon (Pd/C) or disodium tetrachloropalladate (Na_2PdCl_4) was added to the optimized model reaction at 0.3 mM in place of *Da*PdNPs. The reactions were conducted and analyzed by following the protocol in Section S4.

Table S2 *Coupling reaction in the presence of other Pd catalysts*

#	Ar-X (30 mM)	Ar-C≡CH (60 mM)	Pd catalyst	Ligand	Base	Product	
						%conversion	±SD
1	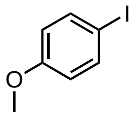	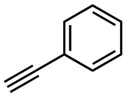	*Da*PdNPs	JohnPhos	K_2CO_3	94	0.58
2			*c*PdNPs			N.D.	N.A.
3			Pd/C			43	8.6
4			Na_2PdCl_4			92	1.0

N.D.: Not detected, N.A.: Not applicable. Product concentrations were determined by ^{1}H NMR relative to an internal standard of TMB (10 mM).

S7 Negative control experiments

Negative control experiments were performed in the optimized model reaction (*Da*PdNPs (0.25 mM), 4-bromoanisole/4-iodoanisole (25 mM), phenylacetylene (30 mM) and K_2CO_3 (30 mM)) in the presence/absence of JohnPhos (2.5 mM) or TPGS-1000 (2% w/vol). The reactions were conducted and analyzed by following the protocol in Section S4.

Table S3 *Effect of JohnPhos and TPGS-1000 on reaction yield*

#	Ar-X (25 mM)	Ar-C≡CH (30 mM)	Ligand	Additive	Product	
					%conv.	±SD
1	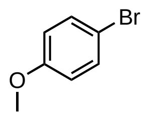	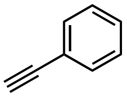	−	−	1	0.12
2			−	TPGS-1000	2	0.20
3			JohnPhos	−	9	0.85
4			JohnPhos	TPGS-1000	20	2.5
5	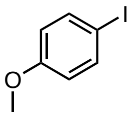		−	−	9	0.85
6			−	TPGS-1000	9	0.09
7			JohnPhos	−	48	1.5
8			JohnPhos	TPGS-1000	99	2.8

Product concentrations were determined by ^{1}H NMR relative to an internal standard of TMB (10 mM).

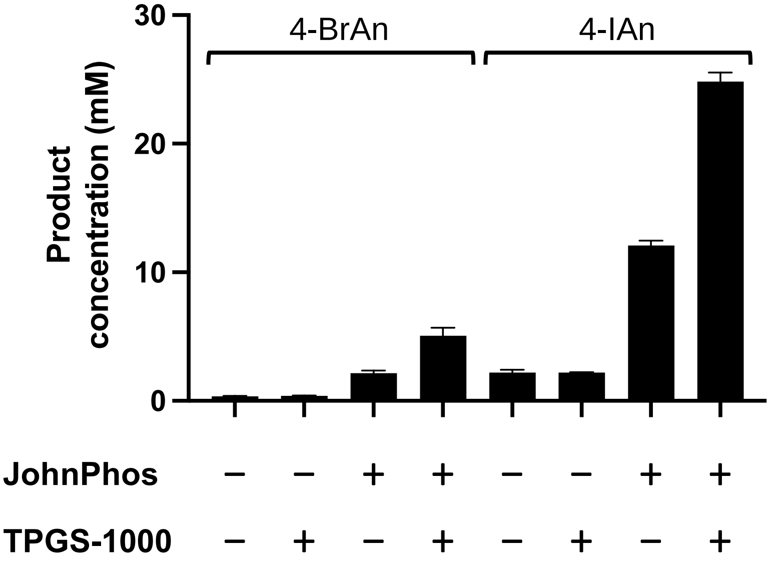

Figure S1. *Effect of JohnPhos and TPGS-1000 on reaction yield (+: presence, −: absence).* Substrate was added at 25 mM.

S8 Reaction scope investigation of Sonogashira coupling

Aryl iodide (4-iodoacetophenone, 3-iodoacetophenone, 1-iodo-4-nitrobenzene or 4-iodophenol: 25 mM) and alkyne (4-ethynyltoluene, 3-ethynylpyridine, 4-ethynylanisole, 3-ethynylanisole, 2-ethynylanisole or trimethylsilylacetylene : 30 mM) were added to a Hungate tube containing *Da*PdNPs (0.25 mM) and K_2CO_3 (30 mM) with/without TPGS-1000 (2% w/vol) in H_2O (5 mL). Reactions of 24 different cross-coupling combinations were carried out and analyzed as outlined in Section S4.

Table S4 *Reaction scope of Sonogashira reaction*

#	Ar-X (25 mM)	R-C≡CH (30 mM)	Ligand	*Da*PdNPs loading (mol%)	Additive	Product	
						%conv.	±SD
1	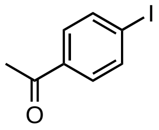	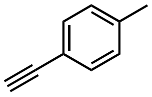	JohnPhos	1.0	–	61	11.6
2					TPGS-1000	99	1.1
3		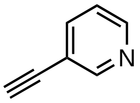			–	44	4.9
4					TPGS-1000	51	1.2
5		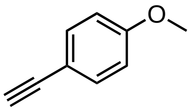			–	41	3.2
6					TPGS-1000	91	1.3
7		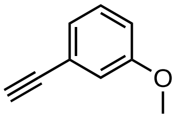			–	29	6.0
8					TPGS-1000	91	0.55
9		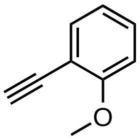			–	44	1.8
10					TPGS-1000	71	3.4
11		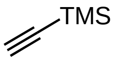			–	5	1.7
12					TPGS-1000	10	0.41
13	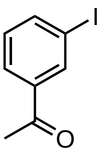	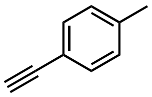	JohnPhos	1.0	–	56	3.7
14					TPGS-1000	98	0.69
15					–	46	5.3

16		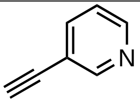			TPGS-1000	54	2.3
17		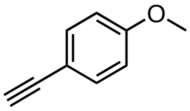			–	52	2.1
18					TPGS-1000	92	3.6
19		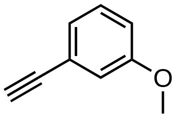			–	52	2.0
20					TPGS-1000	93	3.2
21		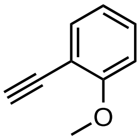			–	45	2.7
22					TPGS-1000	72	4.2
23		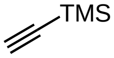			–	7	0.90
24					TPGS-1000	18	2.0
25	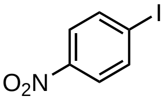	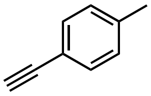	JohnPhos	1.0	–	22	1.5
26					TPGS-1000	76	1.8
27		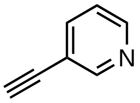			–	7	1.6
28					TPGS-1000	41	1.3
29		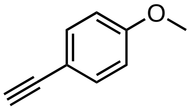			–	27	1.9
30					TPGS-1000	82	1.6
31		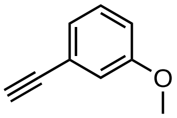			–	25	2.4
32					TPGS-1000	80	2.6
33		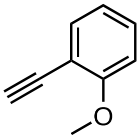			–	22	7.6
34					TPGS-1000	64	1.9
37	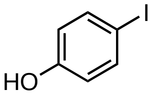	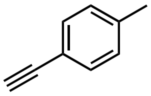	JohnPhos	1.0	–	12	0.57
38					TPGS-1000	37	0.67
39		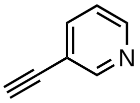			–	9	1.4
40					TPGS-1000	14	1.0
41		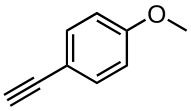			–	16	2.5
42					TPGS-1000	33	4.4

43		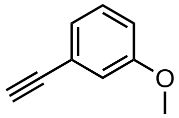			–	16	2.6
44					TPGS-1000	43	0.80
45		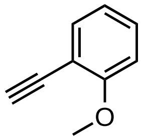			–	8	0.84
46					TPGS-1000	13	0.72

Product concentrations were determined by ^{1}H NMR relative to an internal standard of TMB (10 mM).

S9 Base-free Sonogashira coupling reaction

The optimized model reaction was performed in different media (H_2O, PBS or M9-glucose) in the absence of base. The reactions were conducted and analyzed by following the protocol in Section S4. Subsequently, the reaction was repeated in M9-glucose using a lower concentration of starting materials in the presence of 0.05 mM or 0.25 mM *Da*PdNPs.

Table S5 *Base-free Sonogashira coupling reaction*

#	Ar-X	Ar-C≡CH	*Da*PdNPs loading (mol%)	Base	Reaction media	Product	
						%conv.	±SD
1	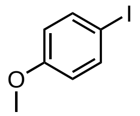 (25 mM)	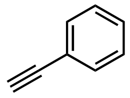 (30 mM)	1.0	–	H_2O	N.D.	N.A.
2					PBS	12	1.5
3					M9-glucose	18	1.0
4	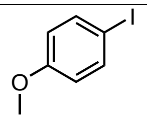 (5 mM)	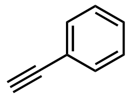 (6 mM)				53	10.0
5			5.0			80	2.9

N.D.: Not detected, N.A.: Not applicable. Product concentrations were determined by ^{1}H NMR relative to an internal standard of TMB (10 mM).

S10 Media screen for biocompatible Sonogashira coupling reaction

For biocompatible reactions, iodobenzene (1 mM) was used in place of 4-iodoanisole. The reactivity of the Sonogashira reaction with iodobenzene was examined using the optimal condition with base (phenylacetylene (1 mM), *Da*PdNPs (0.25 mM), JohnPhos (2.5 mM), K_2CO_3 (30 mM) and TPGS-1000 (2% w/vol) in 5 mL H_2O). The reactions were incubated at 37 °C (200 rpm) for 20 h. After this time, to a 2 mL Eppendorf tube containing 0.45 mL brine and 0.05 mL HCl (1 M), an aliquot (0.5 mL) of the post

reaction mixture was added, and the mixture was extracted with $CDCl_3$ containing 2 mM TMB (2 x 0.5 mL) by vortexing and centrifugation. The combined organic layers were dried over sodium sulfate and analyzed by ^{1}H NMR spectroscopy. Subsequently, the identical reaction in the base-free growth media (M9-glucose, M9CA-glucose, M9-glucose (+ 10% CA) or MM1) was performed and analyzed as described above.

Table S6 *Biocompatible Sonogashira coupling reaction*

#	Ar-X (1 mM)	Ar-C≡CH (1 mM)	Base (eq.)	Reaction media	Product	
					%conv.	±SD
**1**	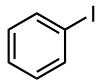	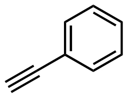	K_2CO_3 (30.0)	H_2O	>99	0.3
**2**			–	M9-glucose	80	12.2
**3**				M9CA-glucose	N.D.	N.A.
**4**				M9-glucose +10% CA	73	2.2
**5**				MM1	81	7.5

N.D.: Not detected, N.A.: Not applicable. Product concentrations were determined by ^{1}H NMR relative to an internal standard of TMB (2 mM).

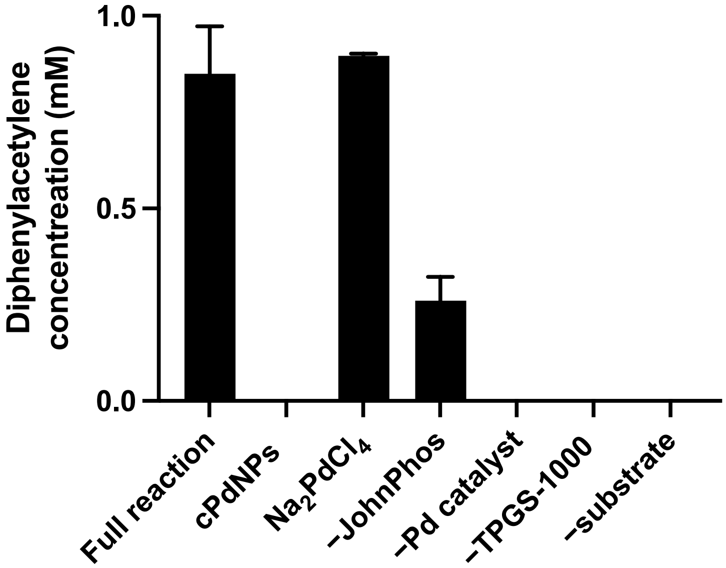

Figure S2. *Control experiments – biocompatible Sonogashira reaction. Full reaction [DaPdNPs (0.25 mM), iodobenzene (1 mM), phenylacetylene (1 mM), JohnPhos (2.5 mM) and TPGS-1000 (2% w/vol) in M9-glucose +10% CA incubated at 37 °C (200 rpm) for 44 h].* *c*PdNPs: chemically produced Pd nanoparticles.

S11 Media screen for bio-hydrogenation of diphenylacetylene

E. coli DD-2 was pre-grown aerobically overnight in the growth media (+ antibiotics) used for the following hydrogenation reactions (M9-glucose, M9CA-glucose, M9-glucose (+ 10% CA) or MM1). The overnight culture was then diluted 1:100 into an appropriate volume of media containing antibiotics, and incubated at 37 °C (200 rpm) until the OD_{600} reached 0.5–0.6.

To a 15 mL Hungate tube containing 5 mL *E. coli* DD-2 culture, *Da*PdNPs (0.25 mM), diphenylacetylene (1 mM), IPTG (0.5 mM), $Fe(NH_4)_2(SO_4)_2$ (0.05 mM) and TPGS-1000 (2% w/vol) were added. The tubes were sealed with butyl rubber septa and screw-caps. The reactions were sparged with nitrogen gas for 10 min using a 21-gauge, 4.25-inch needle as the inlet and a 25-gauge, 0.63-inch needle as the outlet. The reactions were incubated at 37 °C (200 rpm) for 20 h or 44 h. The reaction was processed and analyzed by following the protocol in Section S10.

Table S7 *Bio-hydrogenation reaction*

#	Starting material (1 mM)	Rxn time (h)	Reaction media	*E*-STB		*Z*-STB		BB	
				%conv.	±SD	%conv.	±SD	%conv.	±SD
1	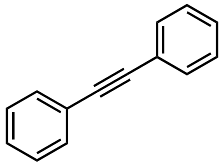	20	M9-glucose	N.D.	N.A.	N.D.	N.A.	N.D.	N.A.
2		20	M9CA-glucose	8	2.1	50	2.1	N.D.	N.A.
3		44	M9-glucose +10% CA	24	2.8	13	9.2	60	1.4

N.D.: Not detected, N.A.: Not applicable, *E*-STB: *trans*-stilbene, *Z*-STB: *cis*-stilbene, BB: bibenzyl. Product concentrations were determined by ^{1}H NMR relative to an internal standard of TMB (2 mM).

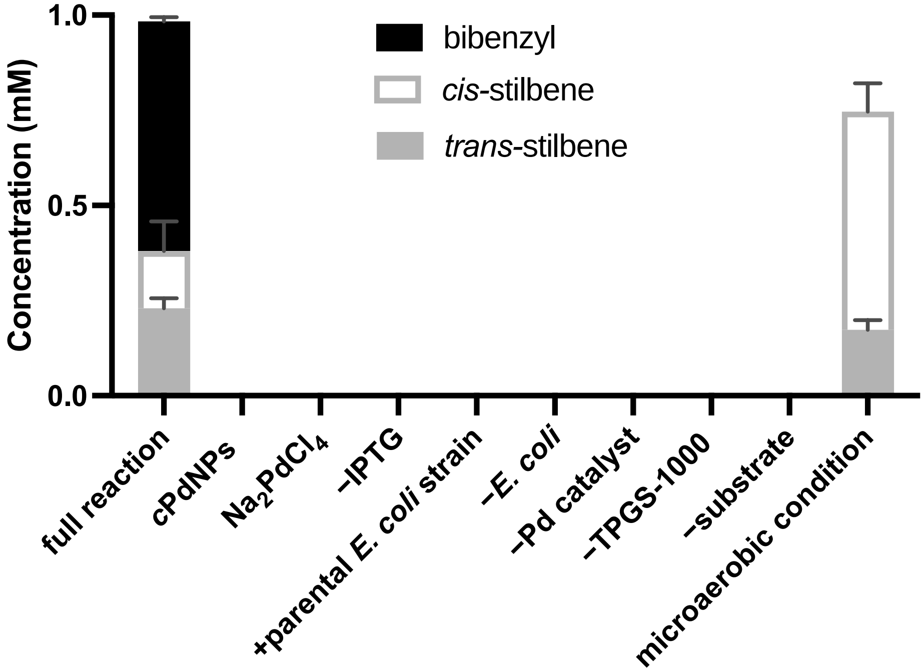

Figure S3. *Control experiments – bio-hydrogenation reaction. Full reaction [DaPdNPs (0.25 mM), diphenylacetylene (1 mM), IPTG (0.5 mM), $Fe(NH_4)_2(SO_4)_2$ (0.05 mM) and TPGS-1000 (2% w/vol) in E. coli DD-2 culture in M9-glucose +10% CA containing antibiotics incubated at 37 °C (200 rpm) for 44 h].*

S12 Sonogashira/hydrogenation cascade reaction in two tube system

Reaction A containing *Da*PdNPs (0.5 mM), iodobenzene (2 mM), phenylacetylene (2 mM), JohnPhos (5 mM) and TPGS-1000 (2% w/vol) in 6.25 mL M9-glucose +10% CA was sealed in a Hungate tube with butyl rubber septa and a screw-cap. The reaction mixture was sparged with nitrogen gas for 10 min and incubated at 37 °C (200 rpm). After 24 h, to an anaerobic culture tube (18 x 150 mm, Chemglass Life Science), **Reaction B** containing pre-grown *E. coli* DD-2 (OD_{600} = 0.5), IPTG (0.5 mM), $Fe(NH_4)_2(SO_4)_2$ (50 µM) and antibiotics (ampicillin (50 µg/mL), spectinomycin (25 µg/mL) and chloramphenicol (12.5 µg/mL)) in 6.25 ml M9-glucose +10% CA was added. The tube was sealed with a chlorobutyl stopper and an aluminum seal. The reaction was sparged with nitrogen gas for 10 min and incubated at 37 °C (200 rpm) overnight. After 44 h **Reaction A** was initiated, **Reaction A** in the Hungate tube was quickly transferred to the anaerobic culture tube containing **Reaction B** using a 10 mL cylinder and a needle. The combined reaction in the anaerobic culture tube was further incubated at 37 °C (200 rpm) for 48 h. The reaction was processed and analyzed by following the protocol in Section S10.

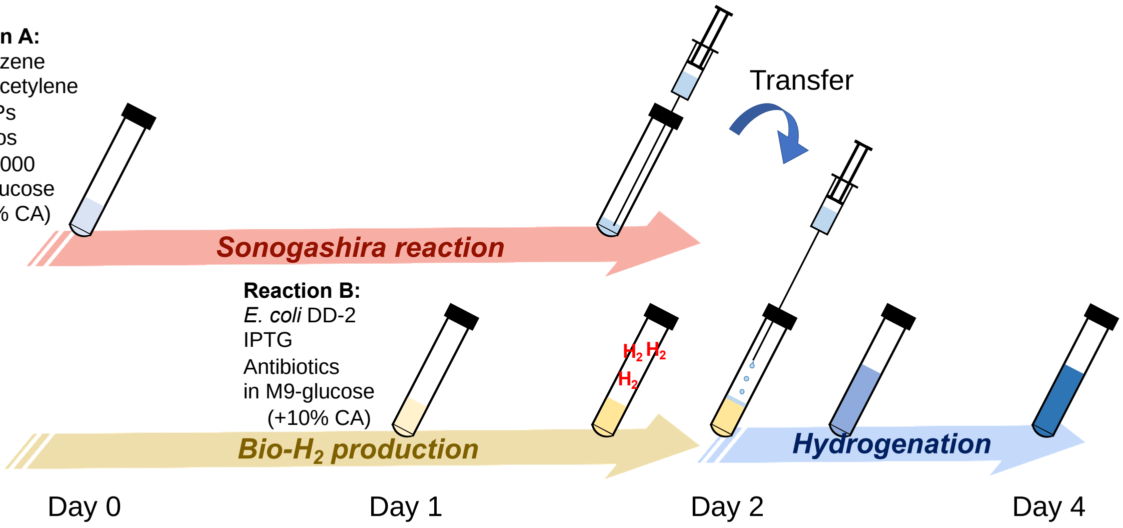

Figure S4. *Scheme of Sonogashira/hydrogenation cascade reaction in two tube system.*

Table S8 *Sonogashira/hydrogenation cascade reaction in two tube system*

#	Ar-X (1 mM)	Ar-C≡CH (1 mM)	Rxn time (h)	DPA		*E*-STB		*Z*-STB		BB	
				%conv.	±SD	%conv.	±SD	%conv.	±SD	%conv.	±SD
1	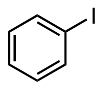	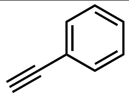	44	78	0.58	N.D.	N.A.	N.D.	N.A.	N.D.	N.A.
2			92	N.D.	N.A.	18	0.0	3	5.0	84	8.1

N.D.: Not detected, N.A.: Not applicable, DPA: diphenylacetylene, *E*-STB: *trans*-stilbene, *Z*-STB: *cis*-stilbene, BB: bibenzyl. Product concentrations were determined by ^{1}H NMR relative to an internal standard of TMB (2 mM).

S13 One-pot Sonogashira/hydrogenation tandem reaction

To a 15 mL Hungate tube containing M9-glucose +10% CA, *Da*PdNPs (0.25 mM), iodobenzene (1 mM), phenylacetylene (1 mM), JohnPhos (2.5 mM) and TPGS-1000 (2% w/vol) were added. The tube was sealed with a butyl rubber septa and a screw-cap and the reaction was sparged with nitrogen gas for 10 min using a 21 gauge, 4.25 inch needle as the inlet and a 25 gauge, 0.63 inch needle as the outlet. The reaction was incubated for 44 h at 37 °C (200 rpm). On Day 2, *E. coli* DD-2 was grown in M9-glucose +10% CA containing antibiotics until the OD_{600} reached 0.5. The cells were pelleted by centrifugation (5 min, 4,500 × g), resuspended in fresh M9-glucose +10% CA to OD_{600} = 12 and 0.5 mL of the suspension was injected into the Hungate tube at 44 h. At the same time, 0.5 mL of M9-glucose +10% CA containing IPTG (12 mM), $Fe(NH_4)_2(SO_4)_2$ (1.2 mM), ampicillin (600 µg/mL), spectinomycin (300 µg/mL) and chloramphenicol

(150 µg/mL) was added to the tube and further incubated at 37 °C (200 rpm) for 5 days. After the reaction plateaued at 164 h (Day 7), fresh *Da*PdNPs (0.25 mM) were added to the tube and incubated until Day 9. Samples were collected every 24 h after 20 h on Day 1, processed and analyzed by following the protocol in Section S10.

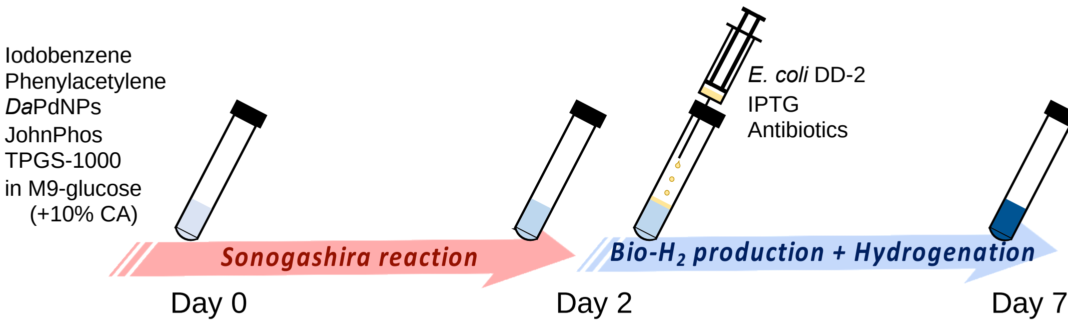

Figure S5. *Scheme of one-pot Sonogashira/hydrogenation tandem reaction.*

Table S9 *One-pot Sonogashira/hydrogenation tandem reaction*

#	Ar-X (1 mM)	Ar-C≡CH (1 mM)	Rxn time (h)	DPA		*E*-STB		*Z*-STB		BB	
				%conv.	±SD	%conv.	±SD	%conv.	±SD	%conv.	±SD
1	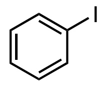	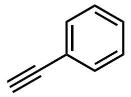	20	61	6.1	N.D.	N.A.	N.D.	N.A.	N.D.	N.A.
2			44	85	12.3	N.D.	N.A.	N.D.	N.A.	N.D.	N.A.
3			68	66	11.6	12	5.2	24	10.4	N.D.	N.A.
4			92	39	5.2	18	2.0	12	5.6	27	6.4
5			116	N.D.	N.A.	19	5.8	17	5.5	52	10.1
6			140	N.D.	N.A.	20	7.0	11	5.0	71	7.1
7			164	N.D.	N.A.	21	3.6	13	4.0	71	9.5

N.D.: Not detected, N.A.: Not applicable, DPA: diphenylacetylene, *E*-STB: *trans*-stilbene, *Z*-STB: *cis*-stilbene, BB: bibenzyl. Product concentrations were determined by ^{1}H NMR relative to an internal standard of TMB (2 or 5 mM).

Table S10 *One-pot Sonogashira/hydrogenation tandem reaction (followed by extra DaPdNPs addition)*

#	Ar-X (1 mM)	Ar-C≡CH (1 mM)	Rxn time (h)	DPA		*E*-STB		*Z*-STB		BB	
				%conv.	±SD	%conv.	±SD	%conv.	±SD	%conv.	±SD
1	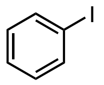	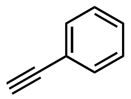	198	N.D.	N.A.	12	0.58	N.D.	N.A.	88	4.6
2			212	N.D.	N.A.	11	3.5	N.D.	N.A.	91	4.4

N.D.: Not detected, N.A.: Not applicable, DPA: diphenylacetylene, *E*-STB: *trans*-stilbene, *Z*-STB: *cis*-stilbene, BB: bibenzyl. Product concentrations were determined by ^{1}H NMR relative to an internal standard of TMB (2 mM).

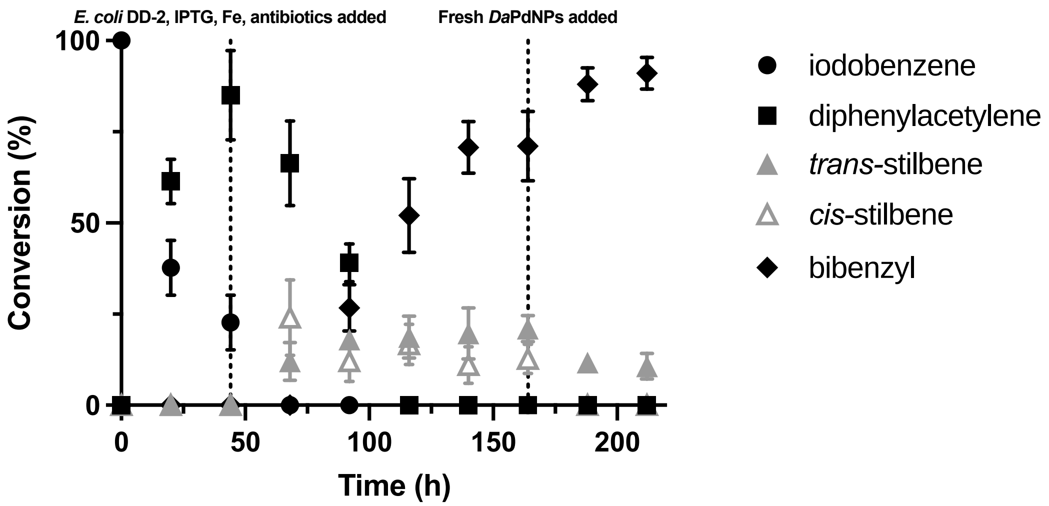

Figure S6. *Reaction time-course.*

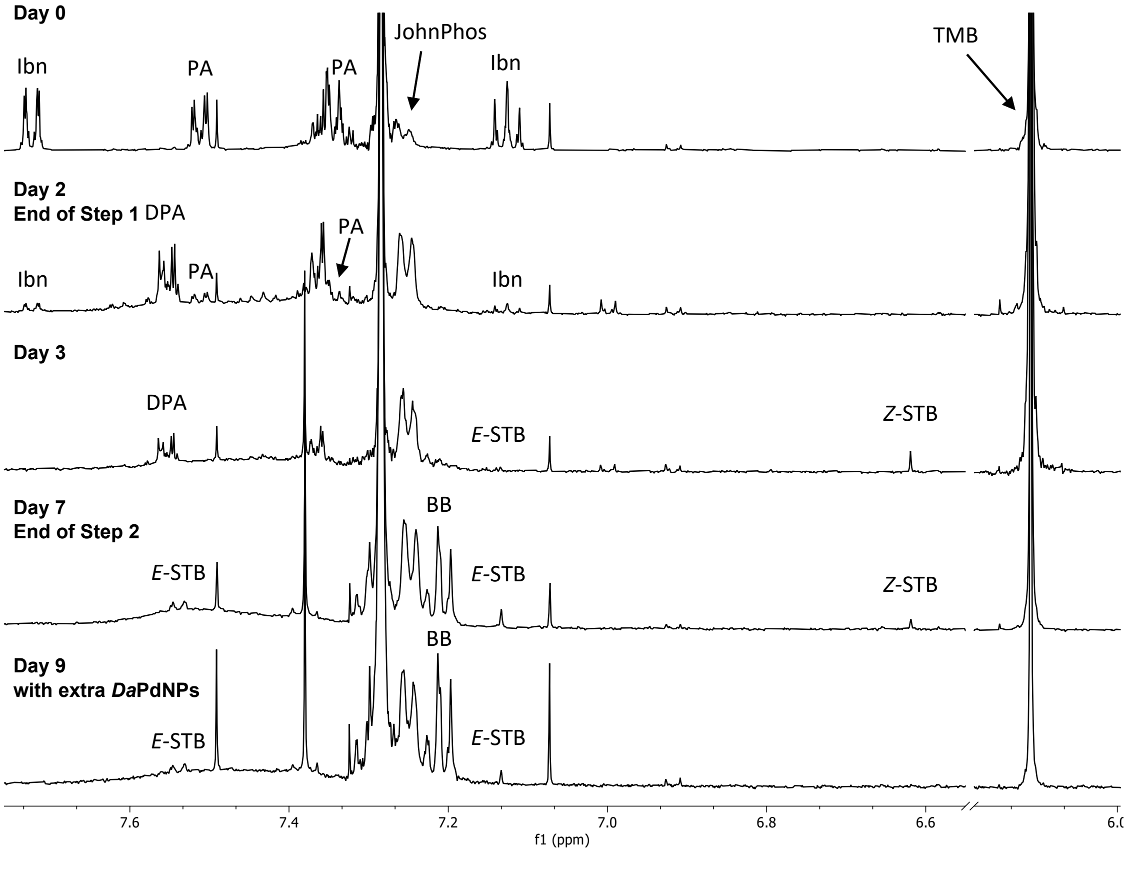

Figure S7. *^{1}H NMR spectra of crude extract of one-pot Sonogashira/hydrogenation tandem reaction at different time points (Ibn: iodobenzene, DPA: diphenylacetylene, E-STB: trans-stilbene, Z-STB: cis-stilbene and BB: bibenzyl).*

S14 Reaction scope investigation of one-pot Sonogashira/hydrogenation cascade

Aryl iodide (4-iodoacetophenone or 3-iodoacetophenone:1 mM) and alkyne (4-ethynyltoluene or 3-ethynylanisole: 1 mM) were added to a Hungate tube containing M9-glucose +10% CA, *Da*PdNPs (0.25 mM), 5 mM K_2CO_3, JohnPhos (2.5 mM) and TPGS-1000 (2% w/v). Reactions were carried out and analyzed as outlined in Section S13.

Table S11 *Reaction scope of Sonogashira/hydrogenation tandem reaction*

#	Ar-X (1 mM)	Ar-C≡CH (1 mM)	Rxn time (h)	DPA derivatives		BB derivatives	
				%conversion	±SD	%conversion	±SD
1	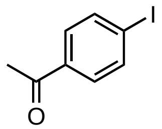	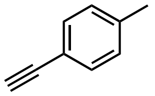	44	85	6.8	N.D.	N.A.
2			164	N.D.	N.A.	72	1.0
3	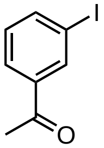	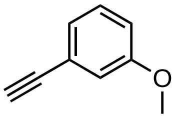	44	90	3.7	N.D.	N.A.
4			164	N.D.	N.A.	76	14.5

N.D.: Not detected, N.A.: Not applicable, DPA: diphenylacetylene, BB: bibenzyl. Product concentrations were determined by ^{1}H NMR relative to an internal standard of TMB (2 mM).

S15 Cell viability assay in Sonogashira/hydrogenation cascade

CFU assay: The one-pot tandem catalysis described in Section S13 was conducted. In addition to the full reaction, a control reaction without *Da*PdNPs, substrates and JohnPhos was performed. At Day 7, 100 µL of each reaction was added to 900 µL of sterile ultrapure water and used to prepare serial dilutions (10^2–10^8). Aliquots (100 µL) of each dilution were plated onto LB agar plates containing ampicillin (50 µg/mL), spectinomycin (25 µg/mL), and chloramphenicol (12.5 µg/mL). The plates were incubated at 37 °C overnight and the plate for the dilution that yielded the highest number of colonies lower than 200 was counted to determine the colony-forming units (CFUs) of the original reactions.

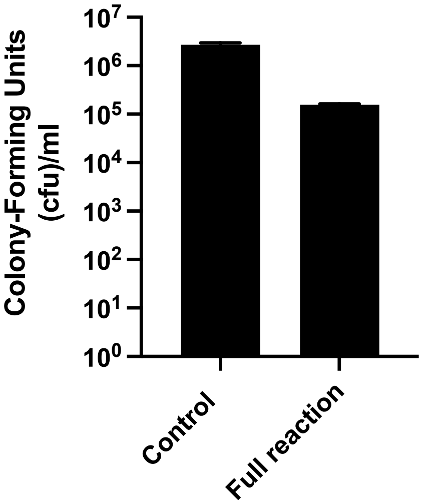

Figure S8. *Plate-count assay. Cultures of E. coli DD-2 in M9-glucose (+10% CA) with/without DaPdNPs, reactants and JohnPhos were incubated anaerobically for 7 days, diluted 10^2-10^8 fold and then plated on LB agar containing antibiotics. CFU values per mL of original culture were determined by counting the number of colonies formed on plate after overnight incubation at 37 ℃.*

Growth curve assay: Bio-hydrogenation reaction in the presence of JohnPhos (2.5 mM) was conducted in M9-glucose +10% CA by following the protocol in Section S11. In addition to the full reaction, –*Da*PdNPs, –substrate/–JohnPhos, control (–*Da*PdNPs/–substrate/–JohnPhos) were performed. Hungate tubes containing identical reactions except for *E. coli* DD-2 were incubated alongside and they were used as blanks to measure the OD_{600} of corresponding inoculated reactions. OD_{600} was monitored until Day 4.

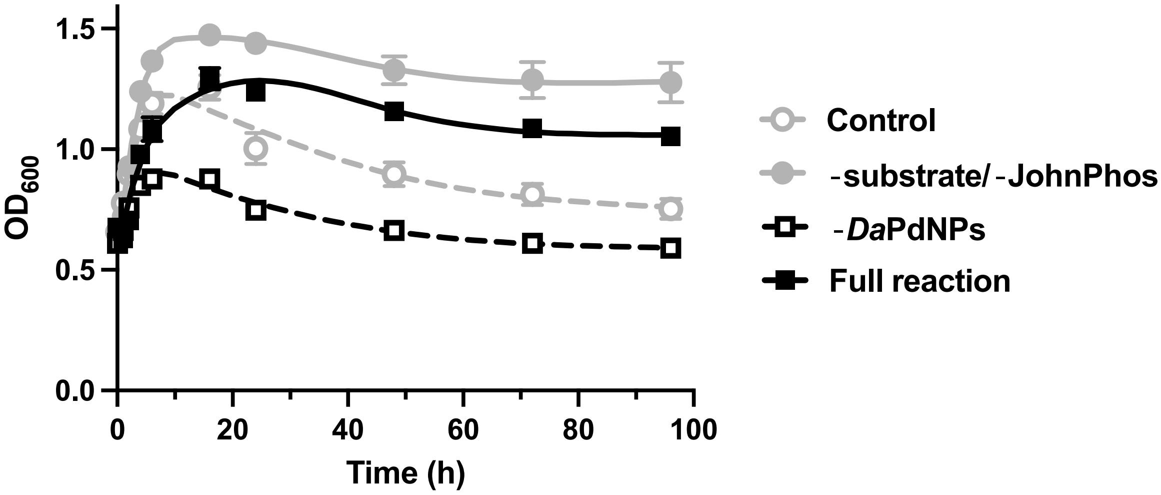

Figure S9. *Growth curve assay.*

S16 Preparative scale reaction

Gram scale Sonogashira reaction from the reaction scope investigation: To a 250 mL flask containing 100 mL H_2O, aryl iodide (4-iodoacetophenone (25 mM, 0.63 g) or 3-iodoacetophenone (25 mM, 0.63 g)), alkyne (4-ethynyltoluene (30 mM, 392 µL) or 3-ethynylanisole (30 mM, 397 µL)), *Da*PdNPs (0.25 mM, 68 mg), JohnPhos (2.5 mM), K_2CO_3 (30 mM) and TPGS-1000 (2% w/vol) were added. The flask was sealed with a Suba-Seal® silicone rubber septa and incubated at 37 °C (200 rpm) for 20 h. After this time, the reaction was cooled to room temperature, extracted with dichloromethane (DCM, 2 x 50 mL), filtered to remove *Da*PdNPs, dried over anhydrous sodium sulfate and concentrated under reduced pressure. The crude product was purified by column chromatography on silica gel (gradient mix of hexane-ethyl acetate) to give 1-{4-[(4-methylphenyl)ethynyl]phenyl}ethenone as an orange solid (0.41 g, 70% isolated yield containing 5% 4-iodoacetophenone as impurity)) / 1-{3-[(3-methoxyphenyl)ethynyl]phenyl}ethenone as an off-white solid (0.60 g, 96% isolated yield).

Gram scale one-pot Sonogashira/hydrogenation cascade: To a 4 L flask containing 1.5 L M9-glucose +10% CA, iodobenzene (1 mM, 171 µL), phenylacetylene (1 mM, 165 µL), *Da*PdNPs (0.25 mM), JohnPhos (2.5 mM, 1.02 g) and TPGS-1000 (2% w/vol) were added. The flask was sealed with a Suba-Seal® silicone rubber septa and the reaction was sparged with nitrogen gas for 30 min using a 19 gauge, 12 inch needle as the inlet and a 25 gauge, 8 inch needle as the outlet. The reaction was incubated for 44 h at 37 °C (200 rpm). On Day 2, *E. coli* DD-2 was grown in M9-glucose +10% CA containing antibiotics until the OD_{600} reached 0.5. The cells were pelleted by centrifugation (15 min, 4,500 × g), resuspended in fresh M9-glucose +10% CA to OD_{600} = 16 and 500 mL of the suspension was injected into the flask at 44 h. At the same time, 500 mL of M9-glucose +10% CA containing IPTG (16 mM), $Fe(NH_4)_2(SO_4)_2$ (1.6 mM), ampicillin (800 µg/mL), spectinomycin (400 µg/mL) and chloramphenicol (200 µg/mL) was added to the flask and further incubated at 37 °C (200 rpm) for 5 days. After this time, the reaction was cooled to room temperature, mixed with NaCl (3 M, 286 g), extracted with $CHCl_3$ (2 x 800 mL), filtered to remove *Da*PdNPs, dried over sodium sulfate and concentrated under reduced pressure. The crude product was purified by column chromatography on silica gel (hexane) affording the product mixture shown in Table S11 as clear crystals (0.29 g).

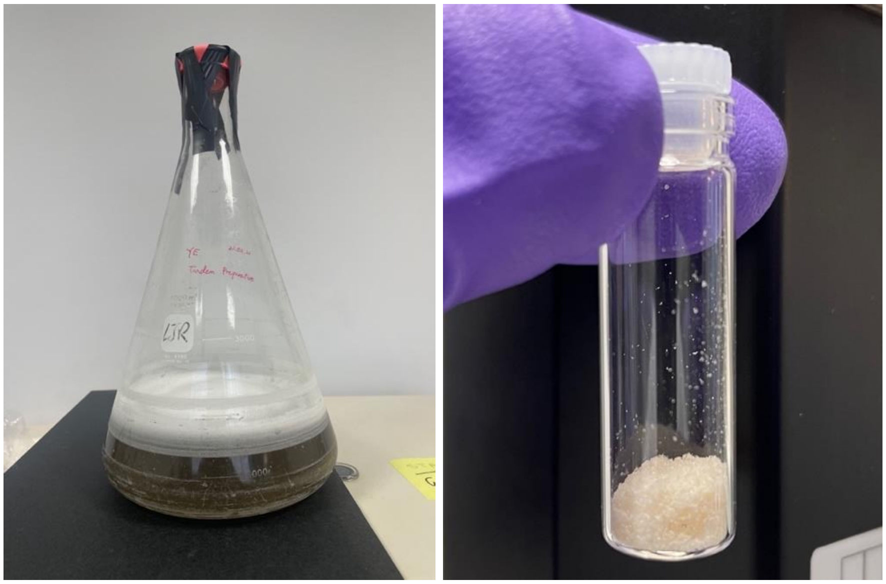

***Figure S10**. Preparative scale one-pot Sonogashira/hydrogenation cascade. The reaction at Day 0 (left) and isolated product mixture (right).*

***Table S12** Composition of isolated product in preparative scale Sonogashira/hydrogenation cascade*

	Isolated yield	Product mass
bibenzyl	66%	0.18 g
cis-stilbene	13%	0.04 g
trans-stilbene	13%	0.04 g
total	92%	0.25 g

Pd content quantification in isolated products: Each isolated product (45 mg of Sonogashira product/3 mg of tandem catalysis product) was dissolved in 1 mL DCM. The organic mixture was extracted with 2% nitric acid (2 x 2.5 mL) and the combined aqueous layer was analyzed by ICP-OES.

***Table S13** Pd content in isolated products from preparative scale reactions*

	Pd concentration (µg/g of product)
1-{4-[(4-methylphenyl)ethynyl]phenyl}ethenone	0.17
1-{3-[(3-methoxyphenyl)ethynyl]phenyl}ethenone	N.D.
bibenzyl	18.7

S17 TEM analysis of *Da*PdNPs, *E. coli* DD-2 and TPGS micelles

To a 15 mL Hungate tube containing 5 mL *E. coli* DD-2 culture (OD_{600} = 0.5), *Da*PdNPs (0.25 mM), and TPGS-1000 (0.4% w/vol) were added. In addition, control samples without TPGS-1000 and or without both *Da*PdNPs and TPGS-1000 were prepared. The tubes were sealed with butyl rubber septa and screw-caps. The reactions were sparged with nitrogen gas for 10 min using a 21-gauge, 4.25-inch needle as the inlet and a 25-gauge, 0.63-inch needle as the outlet. The reactions were incubated at 37 °C (200 rpm) for 12 h. After this time, 100 µL of each reaction was removed and added to 900 µL H_2O, and a drop of each solution was transferred on to a Formvar/Carbon 200 mesh copper grid and left to dry in air for 10 min. Excess solution was removed by touching the grid edge with filter paper. A drop of 1% aqueous uranyl acetate was applied for 1 min and then removed by touching the grid edge with filter paper. TEM observation was carried out as described in Section S1.

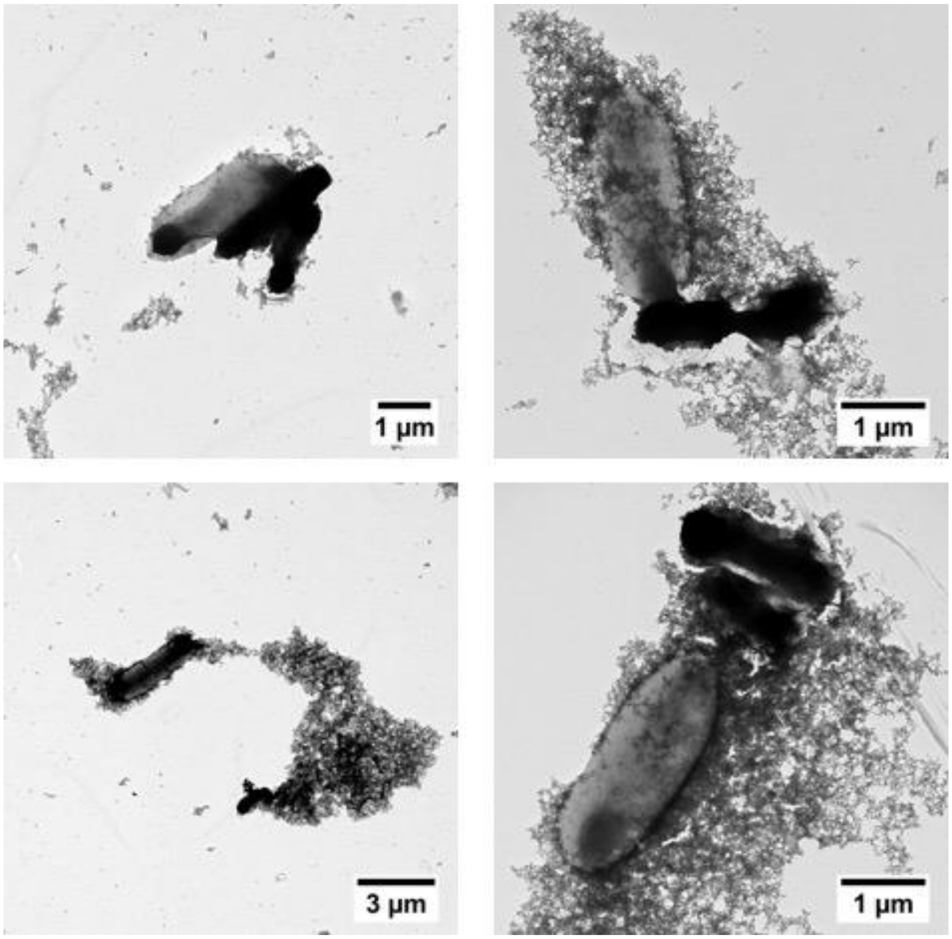

Figure S11. *Negative stained DaPdNPs and E. coli DD-2 in the presence of 0.4% w/vol TPGS-1000.*

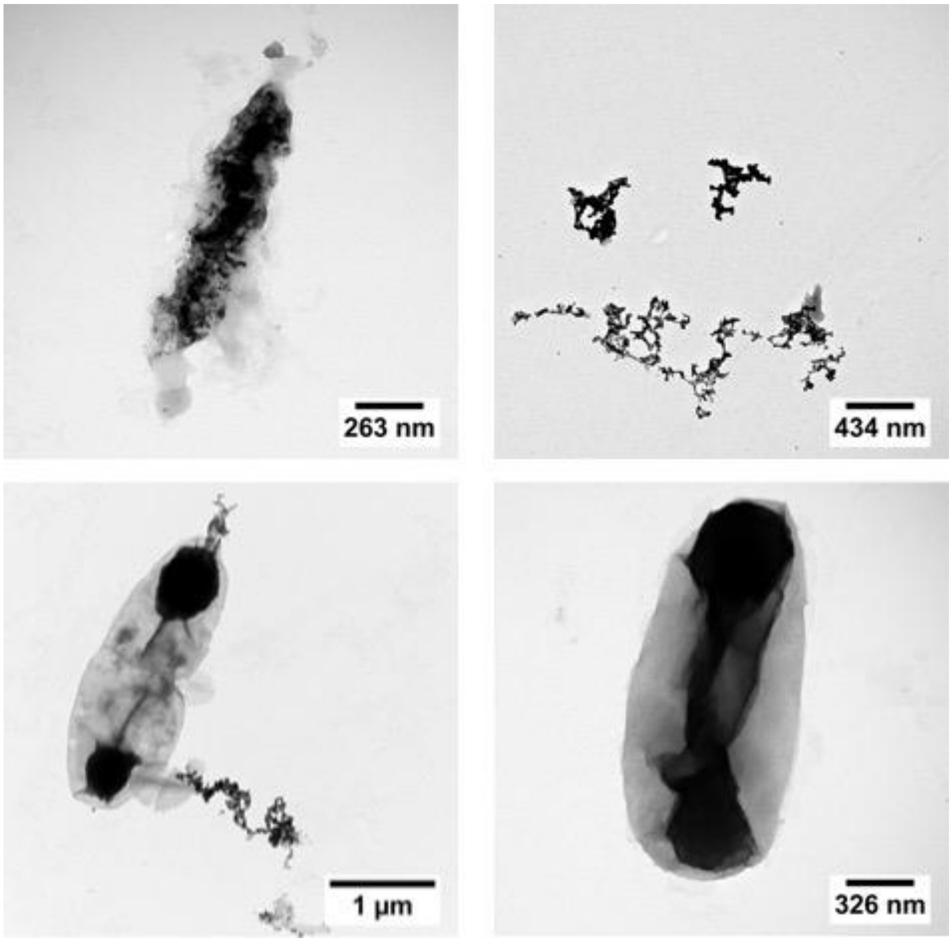

Figure S12. *Negative stained DaPdNPs and E. coli DD-2 in the absence of TPGS-1000.*

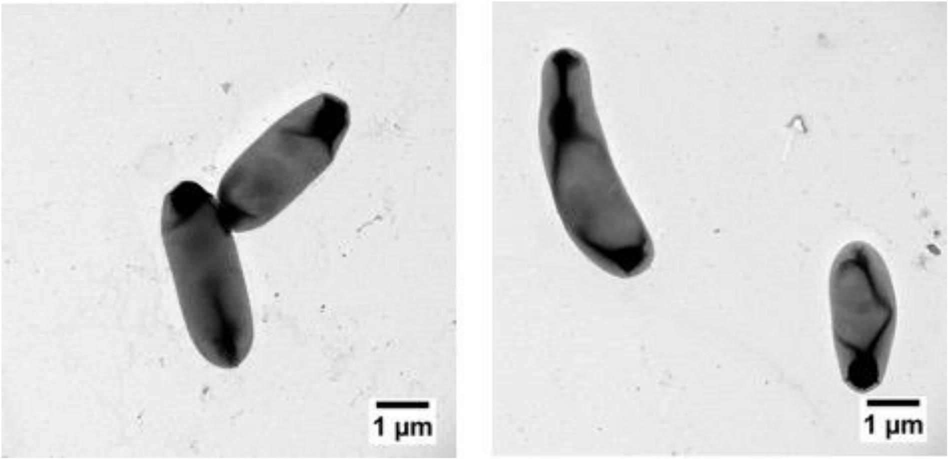

Figure S13. *Negative stained E. coli DD-2.*

S18 Product Characterization

1-Methoxy-4-(phenylethynyl)benzene

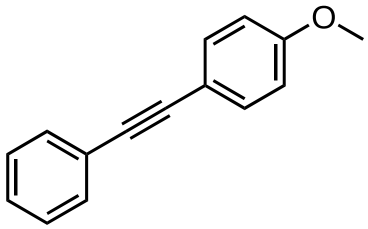

^{1}H NMR (500 MHz, $CDCl_3$) δ: 7.73 – 7.37 (m, 4H), 7.32 (dddd, *J*=8.9, 7.5, 5.8, 1.9, 4H), 6.94 – 6.75 (m, 1H), 3.65 (s, 3H); ^{13}C NMR (126 MHz, $CDCl_3$) δ: 55.3, 88.1, 89.37, 114.0 (2C), 115.4, 123.6, 127.9, 128.3 (2C), 131.5 (2C), 133.1 (2C), 159.6. Spectroscopic data in good agreement with the literature.[3]

1-{4-[(4-Methylphenyl)ethynyl]phenyl}ethenone

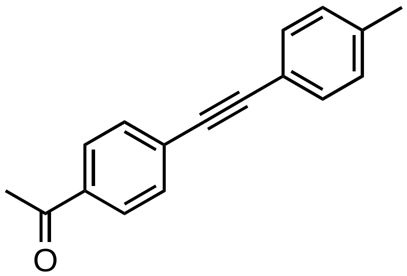

TLC: R_f = 0.24 (silica gel, 1:19 = ethyl acetate : hexane); ^{1}H NMR (500 MHz, $CDCl_3$) δ: 7.94 – 7.89 (m, 2H), 7.61 – 7.55 (m, 2H), 7.46 – 7.40 (m, 2H), 7.19 – 7.14 (m, 2H), 2.59 (s, 3H), 2.37 (s, 3H); ^{13}C NMR (126 MHz, $CDCl_3$) δ: 21.6, 26.61, 88.1, 93.0, 119.6, 128.3 (2C), 128.5, 129.2 (2C), 131.6 (2C), 131.7 (2C), 136.1, 139.1, 197.3; HRMS (EI) found 234.1035 $[M]^{+\bullet}$, $C_{17}H_{14}O^{+\bullet}$ requires 234.1039. Spectroscopic data in good agreement with the literature.[4]

1-{3-[(4-Methoxyphenyl)ethynyl]phenyl}ethenone

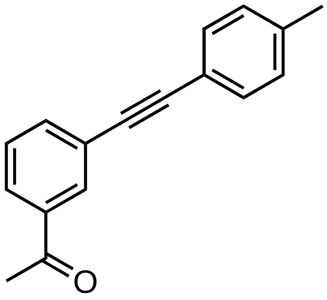

^{1}H NMR (500 MHz, $CDCl_3$) δ: 8.08 (t, *J*=1.7, 1H), 7.89 (ddd, *J*=7.8, 1.8, 1.2, 1H), 7.69 (dt, *J*=7.7, 1.4, 1H), 7.43 (d, *J*=7.9, 3H), 7.18 – 7.14 (m, 2H), 2.61 (s, 3H), 2.37 (s, 3H); ^{13}C NMR (126 MHz, $CDCl_3$) δ: 21.5, 26.7, 87.7, 90.6, 119.7, 124.2, 127.6, 128.7, 129.2 (2C), 131.6, 131.6 (2C), 135.8, 137.2, 138.8, 161.6, 197.5.

1-Methoxy-4-[(4-nitrophenyl)ethynyl]benzene

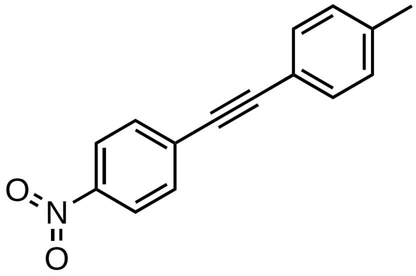

^{1}H NMR (500 MHz, $CDCl_3$) δ: 8.24 – 8.17 (m, 2H), 7.68 – 7.61 (m, 2H), 7.48 – 7.42 (m, 2H), 7.20 (d, *J*=7.8, 2H), 2.39 (s, 3H). Spectroscopic data in good agreement with the literature.[4]

4-[(4-Methoxyphenyl)ethynyl]phenol

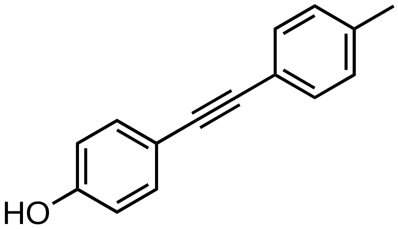

^{1}H NMR (500 MHz, $CDCl_3$) δ: 7.43 – 7.35 (m, 4H), 7.21 – 7.05 (m, 2H), 6.85 – 6.78 (m, 2H), 2.19 (s, 3H). Spectroscopic data in good agreement with the literature.[5]

1-[4-(3-Pyridinylethynyl)phenyl]ethenone

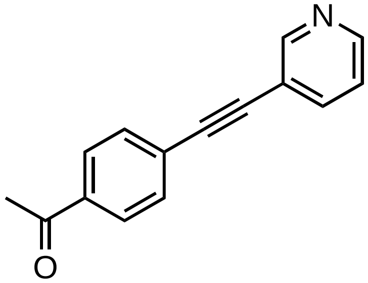

^{1}H NMR (500 MHz, $CDCl_3$) δ: 8.77 (dd, *J*=2.1, 0.9, 1H), 8.57 (dd, *J*=4.9, 1.7, 1H), 7.85 – 7.78 (m, 2H), 7.76 (dt, *J*=7.9, 1.9, 1H), 7.63 – 7.60 (m, 2H), 7.30 (ddd, *J*=7.9, 4.9, 0.9, 1H), 2.60 (s, 3H); ^{13}C NMR (126 MHz, $CDCl_3$) δ: 26.7, 89.1, 91.75, 119.9, 123.1, 127.3, 128.4 (2C), 131.8 (2C), 136.7, 138.6, 149.0, 152.3, 197.4.

1-[3-(3-Pyridinylethynyl)phenyl]ethenone

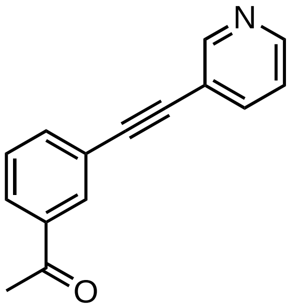

^{1}H NMR (500 MHz, $CDCl_3$) δ: 8.76 (dd, *J*=2.2, 0.9, 1H), 8.55 (dd, *J*=4.9, 1.7, 1H), 8.10 (t, *J*=1.7, 1H), 7.97 – 7.90 (m, 1H), 7.81 (dt, *J*=7.9, 1.9, 1H), 7.71 (dt, *J*=7.7, 1.4, 1H), 7.46 (t, *J*=7.8, 1H), 7.32 – 7.26 (m, 1H), 2.61 (s, 3H); ^{13}C NMR (126 MHz, $CDCl_3$) δ: 26.7, 86.9, 91.54, 120.1, 123.1, 123.2, 128.4, 128.9, 131.7, 135.9, 138.6, 140.5, 148.8, 152.3, 197.2.

3-[(4-Nitrophenyl)ethynyl]pyridine

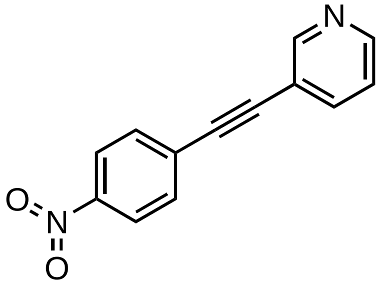

^{1}H NMR (500 MHz, $CDCl_3$) δ: 8.79 (dd, *J*=2.2, 0.9, 1H), 8.61 (dd, *J*=4.9, 1.7, 1H), 8.29 – 8.20 (m, 2H), 7.85 (dt, *J*=7.9, 1.9, 1H), 7.73 – 7.63 (m, 2H), 7.33 (ddd, *J*=7.9, 4.9, 0.9, 1H). Spectroscopic data in good agreement with the literature.[6]

4-(3-Pyridinylethynyl)phenol

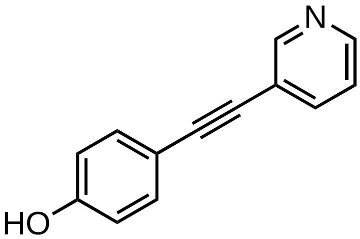

^{1}H NMR (500 MHz, $CDCl_3$) δ: 8.51 (dd, *J*=4.9, 1.7, 1H), 7.90 (dt, *J*=7.2, 1.7, 0H), 7.42 – 7.38 (m, 2H), 7.27 – 7.22 (m, 2H), 6.91 – 6.82 (m, 2H); ^{13}C NMR (126 MHz, $CDCl_3$) δ: 84.3, 93.2, 113.7, 115.8 (2C), 121.1, 123.1, 133.3 (2C), 138.4, 147.9, 151.9, 157.5.

1-{4-[(4-Methoxyphenyl)ethynyl]phenyl}ethenone

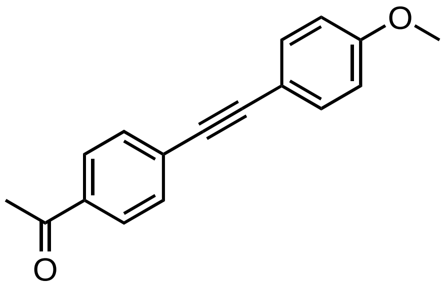

^{1}H NMR (500 MHz, $CDCl_3$) δ: 7.94 – 7.88 (m, 2H), 7.60 – 7.52 (m, 2H), 7.50 – 7.45 (m, 2H), 6.92 – 6.86 (m, 2H), 3.82 (s, 3H), 2.58 (s, 3H). Spectroscopic data in good agreement with the literature.[7]

1-{3-[(4-Methoxyphenyl)ethynyl]phenyl}ethenone

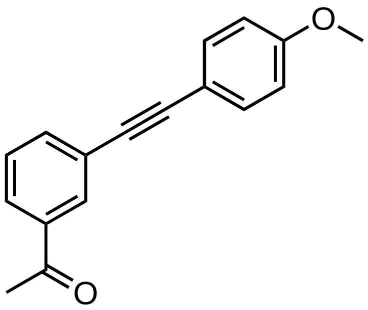

^{1}H NMR (500 MHz, $CDCl_3$) δ: 8.08 (t, *J*=1.7, 1H), 7.88 (ddd, *J*=7.9, 1.9, 1.2, 1H), 7.68 (dt, *J*=7.7, 1.4, 1H), 7.49 – 7.46 (m, 2H), 7.43 (t, *J*=7.7, 1H), 6.91 – 6.86 (m, 2H), 3.82 (s, 3H), 2.61 (s, 3H); ^{13}C NMR (126 MHz, $CDCl_3$) δ: 26.7, 55.3, 87.1, 90.5, 114.1, 114.9 (2C), 124.3, 127.49, 128.7, 131.5, 133.2 (2C), 135.7, 137.2, 159.9, 197.5.

1-Methoxy-4-[(4-nitrophenyl)ethynyl]benzene

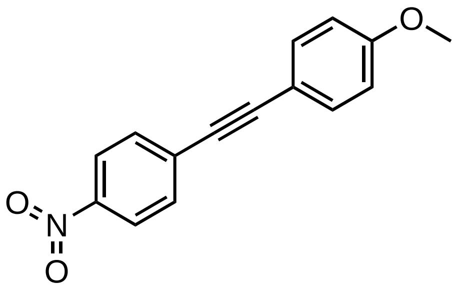

^{1}H NMR (500 MHz, $CDCl_3$) δ: 8.23 – 8.10 (m, 2H), 7.68 – 7.58 (m, 2H), 7.53 – 7.46 (m, 2H), 6.93 – 6.87 (m, 2H), 3.83 (s, 3H). Spectroscopic data in good agreement with the literature.[3]

4-[(4-Methoxyphenyl)ethynyl]phenol

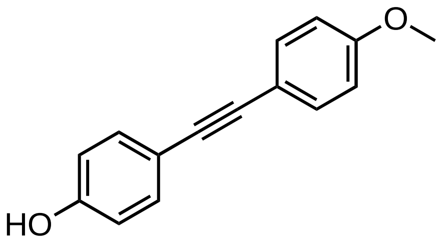

^{1}H NMR (500 MHz, $CDCl_3$) δ: 7.48 – 7.41 (m, 2H), 7.40 – 7.34 (m, 2H), 6.90 – 6.84 (m, 2H), 6.83 – 6.79 (m, 2H), 3.83 (s, 3H). Spectroscopic data in good agreement with the literature.[7]

1-{4-[(3-Methoxyphenyl)ethynyl]phenyl}ethenone

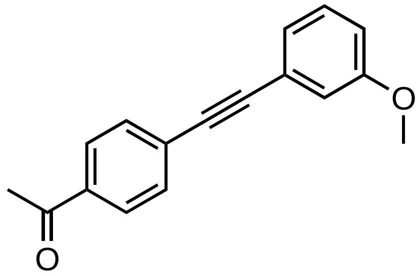

^{1}H NMR (500 MHz, $CDCl_3$) δ: 7.95 – 7.89 (m, 2H), 7.62 – 7.57 (m, 2H), 7.26 (dd, *J*=8.4, 7.6, 1H), 7.13 (dt, *J*=7.6, 1.2, 1H), 7.06 (dd, *J*=2.7, 1.4, 1H), 6.91 (ddd, *J*=8.4, 2.7, 1.0, 1H), 3.82 (s, 3H), 2.59 (s, 3H). Spectroscopic data in good agreement with the literature.[8]

1-{3-[(3-Methoxyphenyl)ethynyl]phenyl}ethenone

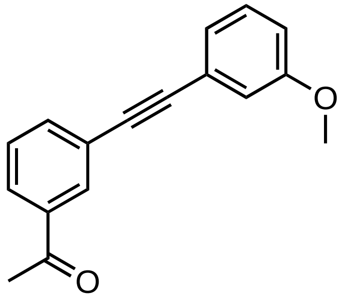

TLC: R_f = 0.15 (silica gel, 1:19 = ethyl acetate : hexane); ^{1}H NMR (500 MHz, $CDCl_3$) δ: 8.10 (t, *J*=1.7, 1H), 7.90 (ddd, *J*=7.8, 1.8, 1.2, 1H), 7.70 (dt, *J*=7.7, 1.4, 1H), 7.45 (t, *J*=7.8, 1H), 7.26 (dd, *J*=8.3, 7.6, 1H), 7.13 (dt, *J*=7.6, 1.2, 1H), 7.06 (dd, *J*=2.7, 1.4, 1H), 6.91 (ddd, *J*=8.4, 2.7, 1.0, 1H), 3.82 (s, 3H), 2.61 (s, 3H); ^{13}C NMR (126 MHz, $CDCl_3$) δ: 26.7, 55.3, 88.1, 90.4, 115.3, 116.5, 123.8, 123.9, 124.3, 127.9, 128.7, 129.5, 131.6, 135.8, 137.3, 159.4, 197.4; HRMS (EI) found 251.1055 $[M+H]^+$, $C_{17}H_{14}O_2+H^+$ requires 251.1066.

1-Methoxy-3-[(4-nitrophenyl)ethynyl]benzene

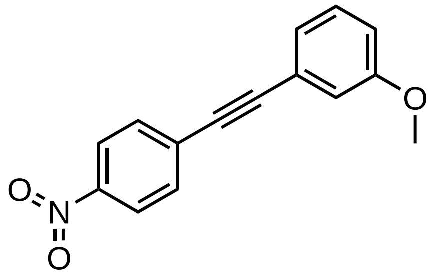

^{1}H NMR (500 MHz, $CDCl_3$) δ: 8.35 – 8.09 (m, 2H), 7.72 – 7.59 (m, 2H), 7.28 (t, *J*=7.9, 1H), 7.15 (dt, *J*=7.5, 1.2, 1H), 7.07 (dd, *J*=2.5, 1.3, 1H), 6.94 (ddd, *J*=8.3, 2.6, 1.0, 1H), 3.82 (s, 3H); ^{13}C NMR (126 MHz, $CDCl_3$) δ: 55.4, 87.3, 94.6, 115.9, 116.6, 123.1, 123.7 (2C), 124.4, 129.6, 130.2, 132.3 (2C), 147.0, 159.5.

4-[(3-Methoxyphenyl)ethynyl]phenol

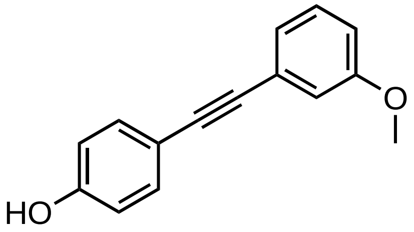

^{1}H NMR (500 MHz, $CDCl_3$) δ: 7.42 – 7.37 (m, 2H), 7.28 – 7.19 (m, 1H), 7.11 (dt, *J*=7.6, 1.2, 1H), 7.05 (dd, *J*=2.7, 1.4, 1H), 6.88 (ddd, *J*=8.3, 2.6, 1.0, 1H), 6.85 – 6.81 (m, 2H), 3.83 (s, 3H); ^{13}C NMR (126 MHz, $CDCl_3$) δ: 55.3, 87.7, 89.4, 114.6, 114.7, 115.4 (2C), 116.2, 124.7, 124.9, 129.4, 133.2 (2C), 156.7, 159.3.

1-{4-[(2-Methoxyphenyl)ethynyl]phenyl}ethenone

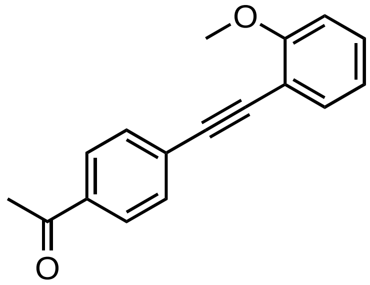

^{1}H NMR (500 MHz, $CDCl_3$) δ: 8.00 – 7.88 (m, 2H), 7.63 – 7.59 (m, 2H), 7.50 (dd, *J*=7.6, 1.7, 1H), 7.37 – 7.30 (m, 1H), 6.95 (td, *J*=7.5, 1.0, 1H), 6.91 (dd, *J*=8.3, 1.0, 1H), 3.91 (s, 3H), 2.60 (s, 3H); ^{13}C NMR (126 MHz, $CDCl_3$) δ: 26.6, 55.3, 89.3, 92.6, 110.8, 111.9, 120.6, 128.2 (2C), 128.6, 130.4, 131.7 (2C), 133.7, 136.1, 160.1, 197.4.

1-{3-[(2-Methoxyphenyl)ethynyl]phenyl}ethenone

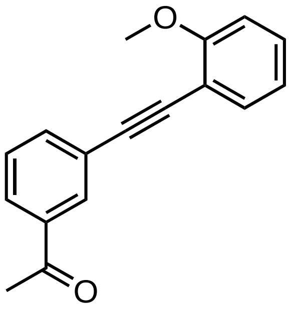

^{1}H NMR (500 MHz, $CDCl_3$) δ: 8.13 (t, *J*=1.6, 1H), 7.90 (ddd, *J*=7.9, 1.8, 1.2, 2H), 7.73 (dt, *J*=7.6, 1.4, 1H), 7.51 (dd, *J*=7.6, 1.7, 1H), 7.45 (d, *J*=7.7, 1H), 7.35 – 7.29 (m, 1H), 6.95 (td, *J*=7.5, 1.0, 1H), 6.92 (dd, *J*=8.4, 1.0, 1H), 3.92 (s, 3H), 2.62 (s, 3H); ^{13}C NMR (126 MHz, $CDCl_3$) δ: 26.7, 55.9, 86.9, 92.3, 110.8, 112.0, 120.6, 124.3, 127.7, 128.6, 130.2, 131.7, 133.7, 135.9, 137.2, 160.1, 197.5.

1-Methoxy-2-[(4-nitrophenyl)ethynyl]benzene

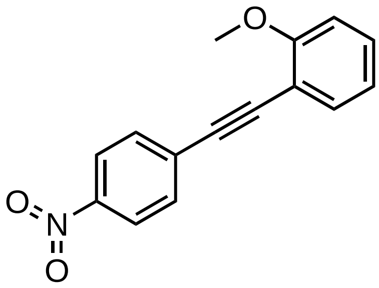

^{1}H NMR (500 MHz, $CDCl_3$) δ: 8.29 – 8.09 (m, 2H), 7.78 – 7.57 (m, 2H), 7.50 (dd, *J*=7.6, 1.7, 1H), 7.36 (ddd, *J*=8.4, 7.5, 1.8, 1H), 6.96 (td, *J*=7.5, 1.0, 1H), 6.93 (dd, *J*=8.6, 1.1, 1H), 3.92 (s, 3H). Spectroscopic data in good agreement with the literature.[9]

4-[(2-Methoxyphenyl)ethynyl]phenol

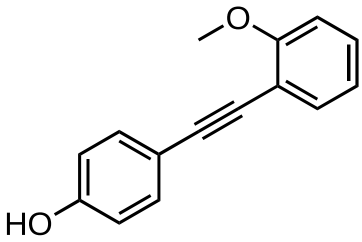

^{1}H NMR (500 MHz, $CDCl_3$) δ: 7.51 – 7.45 (m, 1H), 7.45 – 7.40 (m, 2H), 7.33 – 7.26 (m, 1H), 7.29 – 7.23 (m, 1H), 6.98 – 6.90 (m, 1H), 6.84 – 6.79 (m, 2H), 3.92 (s, 3H); ^{13}C NMR (126 MHz, $CDCl_3$) δ: 55.9, 84.0, 93.6, 110.7, 112.9, 115.1, 115.6 (2C), 120.5, 129.3, 133.2 (2C), 133.4, 156.5, 159.8.

4-[(Trimethylsilyl)ethynyl]acetophenone

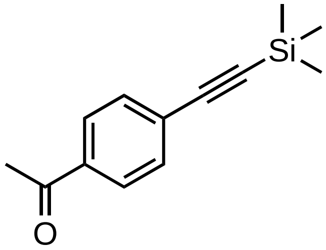

^{1}H NMR (500 MHz, $CDCl_3$) δ: 8.02 – 7.92 (m, 2H), 7.65 – 7.62 (m, 2H), 2.62 (s, 3H), 0.15 (s, 9H). Spectroscopic data in good agreement with the literature.[10]

3-[(Trimethylsilyl)ethynyl]acetophenone

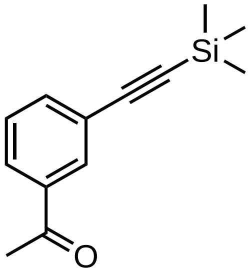

^{1}H NMR (500 MHz, $CDCl_3$) δ: 8.13 (t, *J*=1.6, 1H), 7.95 (ddd, *J*=7.8, 1.8, 1.2, 1H), 7.73 (dt, *J*=7.6, 1.4, 1H), 7.49 (t, *J*=7.7, 1H), 2.64 (s, 3H), 0.15 (s, 9H); ^{13}C NMR (126 MHz, $CDCl_3$) δ: 1.3 (3C), 26.7, 86.8, 89.3, 123.5, 128.2, 128.8, 131.7, 135.9, 137.3, 197.3.

Diphenylacetylene

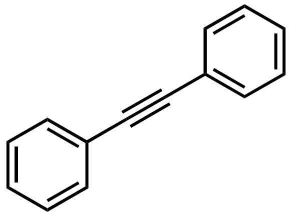

^{1}H NMR (500 MHz, $CDCl_3$) δ: 7.60 – 7.53 (m, 4H), 7.40 – 7.34 (m, 6H). Spectroscopic data in good agreement with the literature.[11]

***(Z)*-stilbene**

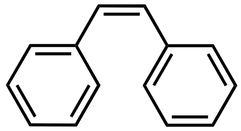

TLC: R_f = 0.32 (silica gel, hexane). ^{1}H NMR (500 MHz, $CDCl_3$) δ: 7.32 – 7.17 (m, 10H), 6.62 (s, 2H). Spectroscopic data in good agreement with the literature.[12]

(E)-stilbene

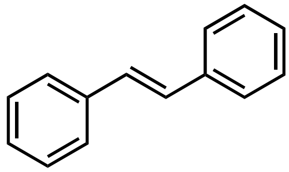

TLC: R_f = 0.24 (silica gel, hexane). ^{1}H NMR (500 MHz, $CDCl_3$) δ: 7.57 – 7.52 (m, 4H), 7.39 – 7.34 (m, 4H), 7.29 – 7.24 (m, 2H), 7.14 (s, 2H). Spectroscopic data in good agreement with the literature.[12]

1,2-Diphenylethane

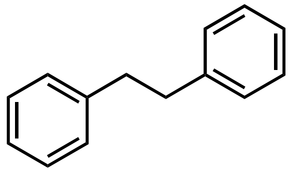

TLC: R_f = 0.23 (silica gel, hexane); ^{1}H NMR (500 MHz, $CDCl_3$) δ: 7.34 – 7.27 (m, 4H), 7.24 – 7.19 (m, 6H), 2.95 (s, 4H); ^{13}C NMR (126 MHz, $CDCl_3$) δ: 38.0 (2C), 125.9 (2C), 128.4 (4C), 128.5 (4C), 141.8 (2C); HRMS (EI) found 182.1084 $[M]^{+\bullet}$, $C_{14}H_{14}^{+\bullet}$ requires 182.1090. Spectroscopic data in good agreement with the literature.[13]

1-(4-Methylphenyl)-2-(4-acylphenyl)ethane

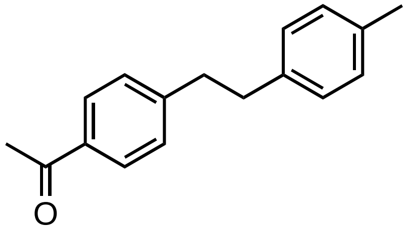

^{1}H NMR (500 MHz, $CDCl_3$) δ: 7.92 – 7.87 (m, 2H), 7.26 – 7.04 (m, 6H), 3.02 – 2.89 (m, 4H), 2.61 (s, 3H), 2.19 (s, 3H); ^{13}C NMR (126 MHz, $CDCl_3$) δ: 21.0, 26.5, 37.0, 38.0, 128.3 (2C), 128.5 (2C), 128.7 (2C), 129.1 (2C), 129.5, 135.6, 138.0, 197.8.

1-(3-Methoxyphenyl)-2-(3-acylphenyl)ethane

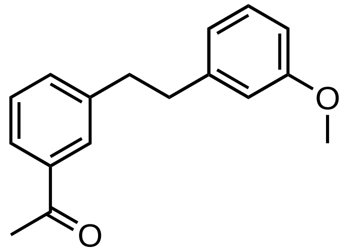

^{1}H NMR (500 MHz, $CDCl_3$) δ: 7.92 (dt, *J*=7.5, 1.7, 1H), 7.83 – 7.77 (m, 2H), 7.25 – 7.20 (m, 1H), 6.81 – 6.70 (m, 4H), 3.80 (s, 3H), 3.04 – 2.90 (m, 4H), 2.60 (s, 3H); ^{13}C NMR (126 MHz, $CDCl_3$) δ: 26.7, 37.6, 37.8, 55.2, 111.4, 114.3, 120.9, 126.2, 128.3, 128.6, 129.4, 133.4, 137.3, 142.2, 142.9, 159.7, 198.4.

S19 References

[1] Agapakis, C. M.; Ducat, D. C.; Boyle, P. M.; Wintermute, E. H.; Way, J. C.; Silver, P. A. Insulation of a synthetic hydrogen metabolism circuit in bacteria. *J. Biol. Eng.*, **2010**, *4*, 3.

[2] Capeness, M. J.; Echavarri-Bravo, V.; Horsfall, L. E. Production of Biogenic Nanoparticles for the Reduction of 4-Nitrophenol and Oxidative Laccase-Like Reactions. *Front. Microbiol.*, **2019**, *10*, 997.

[3] Mori, S.; Yanase, T.: Aoyagi, S.; Monguchi, Y.; Maegawa, T.; Sajiki, H. Ligand-Free Sonogashira Coupling Reactions with Heterogeneous Pd/C as the Catalyst. *Chem. Eur. J.,* **2008**, *14*, 6994.

[4] Gholap, A. R.; Venkatesan, K.; Pasricha, R.; Daniel, T.; Lahoti, R. J.; Srinivasan, K. V. Copper- and ligand-free Sonogashira reaction catalyzed by Pd(0) nanoparticles at ambient conditions under ultrasound irradiation. *J. Org. Chem.*, **2005**, *12*, 4869‒4872.

[5] Comin, M. J.; Czifra, G.; Kedei, N.; Telek, A.; Lewin, N. E.; Kolusheva, S.; Velasquez, J. F.; Kobylarz, R.; Jelinek, R; Blumberg, P. M. ; Marquez, V. E. Conformationally Constrained Analogues of Diacylglycerol (DAG). 31. Modulation of the Biological Properties of Diacylgycerol Lactones (DAG-lactones) Containing Rigid-Rod Acyl Groups Separated from the Core Lactone by Spacer Units of Different Lengths. *J. Med. Chem.*, **2009**, *52*, 3274–3283.

[6] Chelucci, G.; Capitta, F.; Baldino, S. Synthesis of internal alkynes via one-pot palladium-catalyzed and dehydrobromination reactions of 1,1-dibromo-1-alkenes. *Tetrahedron.,* **2008**, *64*, 10250‒10257.

[7] Chandra, M. R.; Oya, S.; Kung, M.; Hou, C.; Jin, L.; Kung, H. F. New Diphenylacetylenes as Probes for Positron Emission Tomographic Imaging of Amyloid Plaques. *J. Med. Chem.,* **2007**, *50*, 2415‒2423.

[8] Sedelmeier, J; Ley, S. V.; Lange, H.; Baxendale, I. R. Pd-EnCat™ TPP30 as a Catalyst for the Generation of Highly Functionalized Aryl- and Alkenyl-Substituted Acetylenes via Microwave-Assisted Sonogashira Type Reactions. *Eur. J. Org. Chem.,* **2009**, *26*, 4412‒4420.

[9] Chen, H. J.; Lin, Z. Y.; Li, M. Y.; Lian, R. J.; Xue, Q. W.; Chung, J. L.; Chen, S. C.; Chen, Y. J. A New, Efficient, and Inexpensive Copper(II)/Salicylic Acid Complex Catalyzed Sonogashira-Type Cross-Coupling of Haloarenes and Iodoheteroarenes with Terminal Alkynes. *Tetrahedron.,* **2010**, *66*, 7755‒7761.

[10] Torborg, C.; Zapf, A.; Beller, M. Palladium Catalysts for Highly Selective Sonogashira Reactions of Aryl and Heteroaryl Bromides. *ChemSusChem.,* **2008**, *1*, 91‒96.

[11] Park, S. B.; Alper, H. Recyclable Sonogashira coupling reactions in an ionic liquid, effected in the absence of both a copper salt and a phosphine. *Chemi. Comm.*, **2004**, *11*, 1306‒1307.

[12] Alonso, F.; Riente, P.; Yus, M. Wittig-Type Olefination of Alcohols Promoted by Nickel Nanoparticles: Synthesis of Polymethoxylated and Polyhydroxylated Stilbenes. *Eur. J. Org. Chem.* **2009**, *34*, 6034‒6042.

[13] Black, P. J.; Edwards, M. G.; Williams, J. M. J. Borrowing Hydrogen: Indirect “Wittig” Olefination for the Formation of C–C Bonds from Alcohols. *Eur. J. Org. Chem.*, **2006**, *19*, 4367‒4378.
